# A study protocol for an mHealth, multi-centre randomized control trial to promote use of postpartum contraception amongst rural women in Punjab, Pakistan

**DOI:** 10.1186/s12884-019-2427-z

**Published:** 2019-08-08

**Authors:** Xaher Gul, Waqas Hameed, Sharmeen Hussain, Ishaque Sheikh, Junaid-ur-Rehman Siddiqui

**Affiliations:** Marie Stopes Society, Plot 21-C, Commercial Area, Old Sunset Boulevard, DHA Phase II, Karachi, Pakistan

**Keywords:** Behaviour change, Family planning, Phone-based intervention, Antenatal care, Postnatal care, Non-inferiority

## Abstract

**Background:**

Provision of family planning services during the immediate post-partum phase is considered effective and cost-efficient for promoting healthy timing and spacing of pregnancies. This research aims to test the effectiveness of mobile phone-based interventions in promoting use of postpartum contraception. Moreover, it will also test the non-inferiority of text and voice messages compared to interactive phone-based counselling.

**Methods:**

A three-arm, 10-month, multicentre, randomized controlled trial will be conducted at 15 social franchise (SF) health facilities in Punjab province of Pakistan. Pregnant women aged 15–44 years who are in their first or second trimester and have a mobile phone for their own use will be eligible to participate in this study. The participants will be randomly allocated to one of three study arms: a) voice and text messages; b) interactive telephone-based counselling; or c) control arm (no additional phone-based support). The intervention counselling module will be developed based on the Integrated Behaviour Model which was recently adapted, and tested for the family planning context in Pakistan. It will broadly cover birth-preparedness, importance of birth spacing, and postnatal care. The phone-based intervention aims to improve women’s ability to use contraception by providing them with information about a range of methods, access to family planning methods through outlets such as Suraj SF providers, connecting them with MSS field health educators to help them reach the centres, motivation by re-enforcing the benefits of contraceptive use on women’s quality of life, and dispelling myths and misconceptions about modern contraceptive methods. Risk differences will be used as the measure of effect of the intervention on the outcomes.

**Discussion:**

The study findings will highlight effectiveness of mobile phone in raising awareness of maternal health and contraception, which in turn, is expected to be translated into increased proportion of: at least four antenatal visits, skilled birth or institutional delivery, postpartum contraceptive use, postnatal check-up, child immunization, and breastfeeding. Moreover, if the text and voice messages approach is proven to be non-inferior to interactive calls, it will provide evidence to making promotion of healthcare less resource intensive, and thereby contribute in improving the efficiency of the healthcare system.

**Trial registration:**

This trial was prospectively registered with the Clinical Trials registry (NCT03612518) on August 2nd, 2018.

**Electronic supplementary material:**

The online version of this article (10.1186/s12884-019-2427-z) contains supplementary material, which is available to authorized users.

## Background

More than 50% of the estimated 303,000 annual maternal deaths globally occur in six developing countries, including Pakistan [1]. Across the globe, efforts to decrease maternal mortality have gained momentum, particularly in light of the Sustainable Goal agenda that states by 2030 no country should have a maternal mortality ratio higher than 70 per 100,000 live births [2]. Further, poor maternal health outcomes have negative implications for newborn and child health. Family planning is a proven, cost-effective way to prevent both maternal and newborn morality [3].

To reduce the risk of adverse maternal, perinatal, and infant outcomes, the World Health Organization (WHO) recommends an interval of at least 24 months between delivery and the subsequent pregnancy (birth-to-pregnancy interval) [4]. Evidence shows that short birth intervals increase the risk of maternal, newborn, infant, and under-5 mortality [5]; and is associated with an increased risk of preterm birth, low birth weight [6], stunting, and underweight children [6].

### Pakistan context

The maternal mortality ratio in Pakistan remains high at 276 per 100,000 live births with only 26% of ever-married women using a modern family planning method despite there being universal awareness of family planning [7], of which 37% discontinue use within 12 months [7]. Reasons for method discontinuation include experiencing side effects or health concerns, unavailability of the method, and lack of access to health services [7]. The current unmet need for family planning is 20%, and it is estimated that satisfying 20% of this unmet need could reduce maternal deaths by 29% annually [7, 8].

Pakistan has one of the highest unmet needs for postpartum family planning among low- and middle-income countries [9]. Though couples’ desire for another child decreases with the birth of each child [7] an alarmingly low proportion of women, 13%, adopt a modern contraceptive method post-partum [7]. It is estimated that the desired number of children is approximately one less than the Total Fertility Rate (TFR) of 3.8 [7].

### Rationale

#### Post-partum contraceptive uptake

Multiple encounters with the health care system during the late ante-natal and immediate post-partum period - and provision of family planning (FP) services during this time are considered effective and cost-efficient methods to promote healthy timing and spacing of pregnancy [10]. To this end, several approaches have been used to interact with women and husbands during either or both antenatal and postnatal periods and through integration with other health service [11–15]. A recent study shows that quality ante-natal services substantially increase the likelihood of postpartum contraceptive uptake. Women are most receptive to advice and counselling regarding FP during the ante-natal and early post-partum period [16] [17],. However, after discharge, most women do not return to health facilities for follow-up visits, presenting a crucial missed opportunity for FP service providers [17]. Due to the high proportion of post-partum women who are lost to follow up, the potential to increase postpartum family planning (PPFP) has not been realized [17].

Although the impact of these interventions is positive, the research is still in nascent stages [9]. There is a dire need for evidence around immediate postpartum family planning services in Asia and Africa [18]. Additionally, to better understand the behavioural indicators that influence the decision to use a modern contraceptive method, there is a need to develop theory-driven interventions [19].

#### Mobile penetration

The declining prices for mobile phones and mobile services and unprecedented increase in mobile penetration [20] [21],, is expected to facilitate the use of mHealth initiatives in resource limited settings [22] [21], [23],. Previous interventions have used mHealth approaches to promote postpartum [23] and post-abortion FP use [24], however, a recent systematic review found no robust evaluations on postpartum FP use. Interventions with robust evaluation methodologies are necessary to establish efficacy of mHealth approaches for increasing postpartum family planning [25].

Mobile penetration in Pakistan is expected to increase from 75 to 95% by 2020 [7]. A recent client exit interview found that approximately two-thirds of Marie Stopes Society (MSS) Social Franchise (SF) clients own a cell phone [26]. High mobile phone ownership presents an opportunity to utilize mHealth approaches to promote postpartum adoption of modern FP methods.

To contribute to current gaps in the literature, PPFP will test whether phone-based support to women during pregnancy and after delivery improves the uptake of immediate postpartum contraception. The mobile health (mHealth) intervention consists of two groups: 1) text and voice messages and 2) interactive phone calls by skilled professionals. Further PPFP explores whether a specific mode of communication is more effective than the other. If text and voice messages are as effective as interactive calls, it presents the opportunity to decrease human resources and make PPFP less resource intensive, consequently increasing scalability and sustainability of the intervention.

### Theoretical framework

**Fig. 1:**
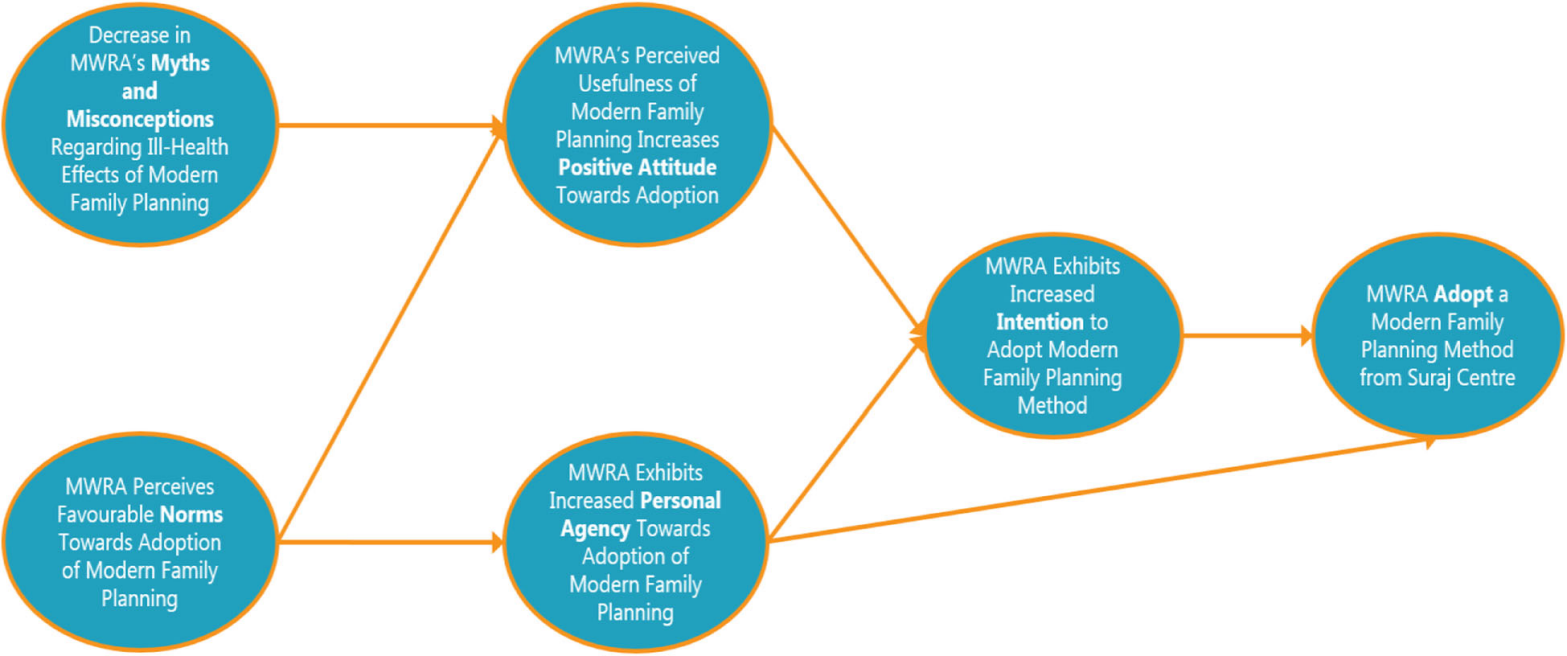
Theoretical Framework

PPFP’s counselling module will be based on the *Integrated Behavior Model* which was recently adapted and validated for modern contraception uptake amongst rural women in Pakistan. This validated model (Fig. 1) shows that the relationship between *Perceived Norms regarding FP* and *Intention to adopt a modern family planning method* is mediated by a *Positive Attitude towards FP* and *Personal Agency to use contraception*. Moreover, the model postulates that women’s attitude towards family planning has two distinct dimensions: 1) myths and misconceptions surrounding modern family planning and 2) women’s belief in the positive impact of modern family planning on their lives or *Positive Attitude*. A woman’s *Positive Attitude* towards family planning is predicted by her *Myths and Misconceptions* and *Perceived Norms* related to modern contraception. Furthermore, *Personal Agency* is also predicted by her *Perceived Norms*. Subsequently, *Personal Agency* and *Positive Attitude* emerge as the two direct predictors of *Intention* to adopt family planning, while *Intention* and *Personal Agency* predict modern contraceptive uptake.

Conceptually, the intervention will aim to improve women’s *capability* to use contraception by providing information about range of methods, *opportunity* to adopt contraception by informing them about *Suraj* SF providers from where they can get services and connecting them with MSS Field Health Educator (FHE) to help them reach the facility, and *motivation* by re-enforcing the benefits of contraceptive use on women’s quality of life, dispelling myths and misconceptions, and providing support for side-effects [27].

## Methods

### Study objectives

#### Primary objective

1. Whether provision of information through structured automated text and voice messages are effective in increasing the use of post-partum contraception;
2. Whether provision of information through structured interactive phone-calls are effective in increasing the use of post-partum contraception; and
3. Whether the automated text & voices messaging approach is as effective as interactive phone-based counseling to increase use of postpartum contraception

#### Secondary objective

1. What are the individual and environmental factors that influence women’s intention to adopt postpartum contraception?

### Study design

PPFP is designed as a three-arm randomized controlled trial across 15 MSS’ social franchise health facilities.

### Study setting

PPFP will be a three-arm, 10-month, multi-centre, randomized controlled trial that is conducted at 15 MSS Suraj social franchise (SF) health facilities, in Punjab province of Pakistan. The target population for this study is pregnant women between 15 and 44 years old who are in their first or second trimester and have access to a mobile phone. Data collection will take place between March 2018 – March 2019 including a three-month recruitment period. FHEs will visit potential respondents at their homes to recruit them for the study. Baseline questionnaires will be administered immediately after recruitment while the endline interview will take place 60 days following childbirth.

#### MSS Suraj social franchise providers

Private providers that have been inducted into the MSS social franchise network and trained for the provision of quality FP services. In addition to these providers, each SF catchment area includes field health educators who conduct meetings and door-to-door visits within the community to promote the uptake of family planning. The MSS social franchise network is comprised of 700 franchises operating in the rural and far flung areas of Sindh, Punjab, and Khyber Pakhtunkhwa provinces, with the catchment area for each provider covering a population of 20,000-25,000.

### Eligibility criteria (inclusion/exclusion)

Married pregnant women with gestational age up to 20 weeks, between 15 and 44 years old, literate i.e. able to read and understand sample family planning messages on the phone, have regular access to a cell phone, and is a permanent resident in the catchment area will be eligible to participate in the study. Women who are not pregnant, or pregnant with gestational age beyond 20 weeks, not between 15 and 44 years of age, illiterate i.e. unable to read or understand sample family planning messages on the phone, does not have regular access to a cell phone, not a permanent resident of the catchment area, and don’t provide consent will not be eligible for the study.

### Interventions

PPFP is a behavior change intervention that aims to improve maternal and neonatal health outcomes by promoting uptake of postpartum contraception. Participants in either of the intervention arms will receive health education and health communication messages that aim to increase postpartum family planning, as well as messages on birth-preparedness and post-natal care. Participants in this study will be randomly assigned to one of three groups: group one will receive this information through text and voice messages; group two through interactive phone calls from a trained professional; while participants assigned to group 3 will not receive phone-based health education messages but can contact their FHE, Suraj provider to the MSS helpline for any information they may need.

#### Health education message development

The development of health education materials will occur in several phases:

1. Researchers will assess the feasibility of an mHealth intervention through in-depth interviews with MSS field health educators who have been working at the selected study sites and with the target population for several years.
2. Researchers will develop FP, birth-preparedness, and post-natal care messages using feedback from FHEs, a validated culturally congruent FP behavior change framework, WHO guidelines, and national guidelines. Further, researchers will seek expert opinions from professionals with experience designing behavior change interventions, training service providers on family planning and safe motherhood, and direct experience working in this field. All messages will be developed in English and later translated to the local language, Urdu.
3. Researchers will pre-test all messages and voice call scripts using cognitive interviews with a subsect of the target population to gather feedback. Additionally, researchers will gather feedback from experts and use findings from both groups to further refine the health education messages.
4. All messages will be tailored to various stages of change for family planning adoption.

Additional information for both intervention arms is included below:

#### Group I: voice and text messages

Participants in this group will receive a mix of voice messages and text messages in the local language. Each participant will receive twenty voice messages over a six-month period (120 days pre-natal and 42 days post-partum) regarding family planning, birth-preparedness and post-natal care. Initially, study participants will receive one voice message a week with the frequency increasing to two messages during the third trimester of the pregnancy and for 42 days following delivery. Postpartum voice messages will consist of a congratulatory message, counselling on maternal and neonatal health needs, breastfeeding, immunization, and planning future pregnancies. Messages will emphasize the need for birth spacing to ensure good health and wellbeing for both the mother and child. Messages will focus on Intra-uterine Contraceptive Devices (IUCDs) because of its effectiveness, high demand [7] and acceptability [28] [29], in social franchise settings.

Similarly, researchers will develop a text message library comprising of messages based on the same themes as the voice messages. Married women of reproductive age (MWRA) will start receiving text messages after the first voice message, at a frequency of two messages per week throughout the intervention period. We will receive read and delivery reports of SMS messages which we will use to build a database of the messages delivered and read by participants. In case of the intervention not yielded an impact, the read reports will be critical in isolating participants who had actually read the messages versus participants who merely received the messages.

Additionally, MWRA will be connected to their respective FHEs and respective Suraj SF providers for additional information and counseling on family planning, birth preparedness, and post-natal care.

#### Group II: interactive phone calls

Participants in this group will receive interactive phone calls from the MSS helpline call centre. These phone calls will be completed by skilled health practitioners (nurses or lady health workers) in the local language. Each participant will receive nine phone calls over a six-month period (120 days pre-natal and 42 days post-partum). Study participants will receive one call every month during the second and third trimester of the pregnancy, one call following delivery and two calls over the next 42 days. Participants will receive health education messages based on the same themes as group one but will have the additional advantage of asking questions to clear any ambiguities. As per standard practice, these calls will be recorded for quality assurance purposes. In case of the intervention not producing an impact, we will build a database by listening to the calls and determine the number of times messages were delivered appropriately for each participant. This will help us in isolating participants to whom messages were appropriately delivered.

Women reporting complications relating to pregnancy, new-born, and contraceptive method use will be referred to nearest public or private health facility.

### Modifications

Researchers will make modifications to the frequency of messages or phone calls during the intervention, if deemed necessary. Researchers will aim to retain participants for follow-up data collection in cases where they discontinue participation in the intervention.

### Adherence/monitoring

The project team will closely monitor the number of messages delivered successfully as well as the number of voice calls attended and the duration of these phone calls. It important to note that a limitation of this study is the inability to track whether the study participant read the text messages or listened to the voice messages until follow-up data collection.

### Concomitant care

The government of Pakistan has a lady health worker (LHW) program operating across the country that also aims to increase contraceptive uptake. However, as participants in the study will be randomized at the individual level, the project team does not expect the LHW program to influence the results of this study. We will capture data on interactions that the participants may have with the LHWs, and subsequently, use it as a control variable in the analysis.

### Outcomes

The following measures will serve as outcomes of interest to evaluate the effectiveness of both interventions:

#### Primary outcome of interest

Action:

1. Proportion of subjects enrolled in each of the three groups who adopt any modern FP method to delay next pregnancy (by type contraceptive method and time within 42 days of postpartum period).
2. Proportion of women reported to be exclusively breastfeeding the newborn
3. Proportion of women reported to immunize the newborn at birth
4. Proportion of women delivered in health facility or skilled birth professional

#### Secondary outcomes of interest

Intention: The proportion of subjects enrolled in the program who state that they intend to adopt any modern FP method to delay their next birth by 24 months (by type contraceptive method and time within 42 days of postpartum period).

Client satisfaction: Score of women’s satisfaction with postpartum family planning services.

#### Process measures

1. MWRA’s modern FP awareness score
2. MWRA’s maternal and newborn health knowledge score of MWRA
3. MWRA’s scores on FP behavior change scales: Perceived norms, myths and misconception, positive attitude, personal agency

### Participant timeline

**Fig. 2:**
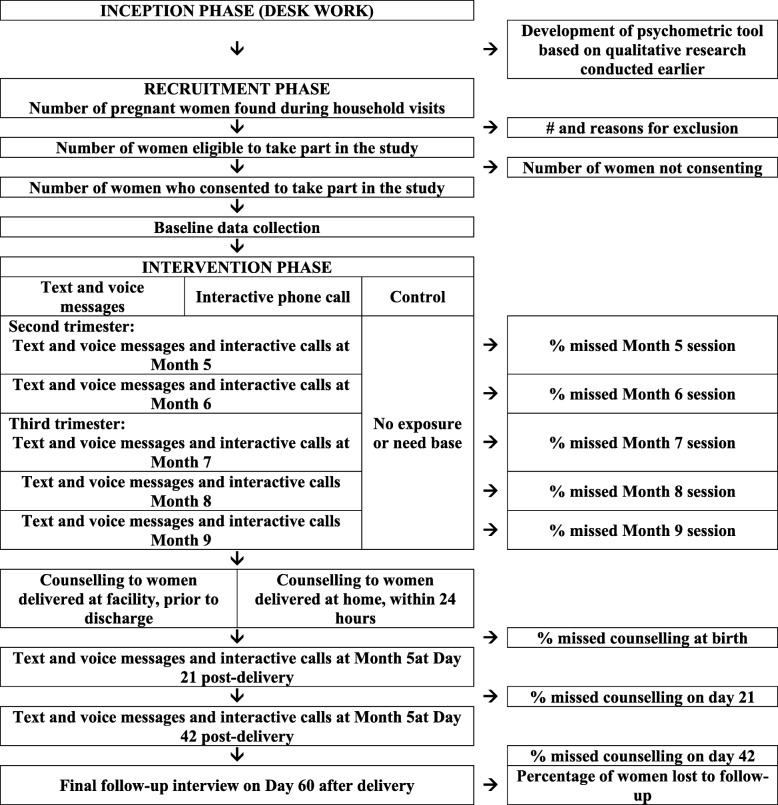
Participant Timeline

The first two months will encompass the inception phase whereby implementation plan for the study will be drafted and finalized, instruments will be finalized, and database will be developed for data collection (see Fig. 2). Months three to five will span the recruitment phase for the study whereby field workers will identify pregnant women, recruit eligibility women into the study, and collect baseline data from them. Months six to twelve span the intervention phase including the final follow-up survey which will be conducted on the 60th day after birth.

### Sample size

An estimated sample size was extracted for each of the research questions. First, we estimated the sample size for the second hypothesis which was based on testing non-inferiority between text/voice messages and interactive phone calls. Non-inferiority tests require relatively higher sample sizes compared to superiority trials. We calculated a sample of 840 (420 in each group) using the following assumption: a non-inferiority margin of ten percentage points (from 25 to 15%) in the use of postpartum contraception within 42 days, 80% power, 0.025 level of significance (one-sided test), 2% missing data/non-response, 8% lost-to-follow-up^1^, and 20% aggregated chance of abortion/miscarriage/fetal loss. We found that an additional sample of 130 sample would be required in the control arm to detect an increase of 15%-points (from 18% [30] to 33%) in the use of PPFP between mobile supported interventions and standard care. This was estimated by using a pre-determined sample of intervention arms (i.e. 420), 0.05 level of significance, while holding other parameters constant. PASS version 11.0 was used for sample size calculation.

### Recruitment

The catchment area of each Suraj SF centre covers a population of approximately 20,000–25,000 [31]. Each centre has an associated field health educator (FHE) who conducts door-to-door visits for family planning counseling [32]. These FHEs will recruit study participants through their routine door-to-door household visits. FHEs will liaise with lady health workers (LHWs), where possible, to help identify eligible participants. To ensure confidentiality and privacy, LHWs will take consent from potential 0participants before disclosing any information to the FHEs for screening and baseline interviews. FHEs will register all eligible women within their catchment areas on a standard data recording format. The final sample will be equally distributed across the study sites (*Suraj* SF centres). Based on vital statistics from Pakistan, it is estimated that recruitment take approximately three to four months.

FHEs will conduct initial eligibility screenings for potential participants during their door-to-door visits. This screening will include the following: age, pregnancy status, self-reported gestational age, and household literacy. If no one in the household, including the potential participant, is literate she will not be considered able to provide informed consent.

Following this screening, FHEs will obtain informed consent from women meeting the pre-consent eligibility criteria. Potential respondents will receive a detailed explanation of the study and their right to participate or refuse participation. FHEs will record all refusals and reasons for refusal. The FHE will give a verbal introduction to the potential participant and will talk them through the written consent form and obtain the participants agreement and signature. We will show a few text messages and play voice messages to the participants for understanding of the exposure to information. The study participant will be offered a copy of the signed consent form to keep for themselves if they wish. A thumb-print consent will be obtained in situations where the woman is not able to provide a signature. If a participant is under 18 years of age, the signed consent form will be taken from the guardian. More specifically, take consent from husband (if available and above 18 years) or mother-in-law or the head of household present at home at the time of interview. We will re-visit if parent or guardian is unavailable during FHE’s visit. We will ensure maintenance of confidentiality for each study participant in order to protect their rights and welfare. All the study participants will be informed about the purpose of study, follow-up mechanism, potential risks and benefits relating to their participation in the study. Study participants will also be given explanation about pregnancy test while taking consent. The result of pregnancy test will not be disclosed to any person other than the respective woman. Moreover, study participants will be reminded and reserve a right to decline answering any question(s) or quit during the interview at any point. All the study participants have the right to choose to partake in the study or refuse to participate and their refusal will not affect their right to MSS services. The study staff will be instructed to record all refusals and to attest to obtaining informed consent from the study participants. The signed consent form refers to all stages of the study (baseline, follow-up counselling, and post-delivery interview at Day-60); however, participants will have a right to withdraw or refuse to participate in the study at any point during the study period. Moreover, before each follow up interview the participants will be offered the opportunity to reread the consent form and the enumerator will check that the participant still agrees to take part in the study.

Women who provide informed consent will be asked to complete a post-consent eligibility assessment including a pregnancy test, access to a mobile phone, and willingness to receive health messages on their mobile phone. Women who meet this eligibility criteria will be enrolled in the study and administered the baseline interview.

### Assignment to intervention or control

Researchers will create a randomization sequence in permuted block sizes of three, six, and nine stratified by the Suraj SF using the *ralloc* command in Stata version 14.1. A specialized computer program (front-end interface) will be developed in Visual FoxPro version 7.0 that will be linked with the randomization database and stored in Stata. This database will be inaccessible to the research assistant who will control the allocation of participants to treatment groups.

After recruiting a participant for the study, FHEs will contact the research assistant by phone for the randomization process. The research assistant will collect preliminary information about the study participant, enter it into the pre-designed software mentioned above, and hit an icon to allocate the participant to a study group. Participants’ information will be sequentially stored in the randomization database. The research assistant will convey the assigned study group to the FHE. To prevent biases, only the principal investigator will have access to the randomization database and will not be involved in the allocation process.

### Data collection

Data will be collected through face-to-face interviews using a structured questionnaire. FHEs will collect participants’ contact information (complete address and telephone number) during recruitment to reach them for the final interviews. This personal data will be stored separately from the interview data.

FHEs will complete data collection at baseline while external enumerators will conduct the final interviews to minimize interviewer biases. All data collectors will undergo comprehensive training on how to approach clients and build rapport; research ethics; data collection; interview techniques; the structured questionnaire; asking and eliciting responses on sensitive topics such as violence; administering and reading pregnancy tests; and the study protocol. Trainers will utilize class-room based training and role play to train data collectors and utilize quizzes affirm comprehension of study procedures.

### Data collection tools

PPFP uses structured questionnaires for data collection at baseline and endline. These questionnaires are expected to take 30–40 min to complete. All data collection tools will be developed in English and later translated to Urdu. The baseline questionnaire will be pre-tested with a cross-section of 20 women from areas comparable to the study sites while FHEs will identify women in their catchment areas who have given birth over the past 12 months to test the follow-up questionnaire. FHEs will obtain consent from women who participate in pre-testing the tools. The tools will be revised based on feedback from pre-testing.

#### Baseline questionnaire

The baseline survey questionnaire is comprised of six sections: a) socio-demographic factors, b) household decision making, c) social support to women, d) reproductive history, e) health seeking behaviors, f) domestic violence, g) knowledge about maternal and newborn health, and h) psychometric assessment regarding contraception. The psychometric tool (section H) will be developed based on finding from a previous qualitative study by MSS with postpartum women. Rest of the sections were previously developed under different studies. Questionnaires in English and Urdu can be found in Additional file 1 and Additional file 3, respectively. FHEs will conduct the baseline interview with participants during their door-to-door visit after completing the eligibility assessment and informed consent process.

Since it is difficult to implement five-point scales in low-literacy settings, we will be using the two-question method. For example, if the scale was “Strongly disagree 1 2 3 4 5 Strongly agree”, the data collector would ask two questions: “Do you agree or disagree?” then “Is your agreement/disagreement complete or partial?” Asking questions in this way will generate the necessary five-point scale in a more comprehensible manner for the population in concern.

#### Endline questionnaire

Endline data collection will take place two months (60 days) after delivery to allow women an additional 15 days after the postpartum period to adopt a contraceptive method as most women are confined to their homes during that time [33]. Prior to the follow-up, enumerators will contact participants to arrange a suitable time and place to conduct the interview. This interview will cover: a) clients’ experiences of childbirth, b) use of essential postpartum and postnatal care; c) contraceptive use and side effects; d) social support during the postpartum period; e) method continuation or reasons for discontinuation or method switching; f) reasons for not adopting contraception, g) pertinent future intentions, and (h) the psychometric assessment. Questionnaires in English and Urdu can be found in Additional file 2 and Additional file 4, respectively. Additionally, enumerators will gather feedback on both interventions from participants.

### Data management

The research team at MSS will be responsible for data management throughout the duration of this study. Researchers will develop the data entry software on Epidata version 3.1 with built-in validation checks to minimize errors in data. Two data operators will complete dual data entry at the MSS head office. Hardcopies of the questionnaires will be sorted according to a unique identifier and stored in assigned cabinets. Enumerators will retain a copy of participant contact sheets that will be used to conduct follow-up visits. Additionally, researchers will maintain a record of (home- or telephone-based) counselling sessions conducted by field health educators and will be monitored during visits by the research team.

### Statistical methods

Demographic and baseline characteristics will be analyzed using descriptive statistics. Central tendency and dispersion including means, medians, standard deviations and range will be calculated for continuous variables. Categorical data will be summarized with frequencies and percentages. A detailed cross-classification analysis will be performed for key outcome indicators by socio-economic factors, health seeking behaviors, household decision making, social support, delivery care, and postpartum contraceptive uptake. Chi-square and t-tests will be used to assess the relationship with risk factors and the outcome variable.

Risk differences will be estimated to test research hypotheses. We will use binomial model (generalized binary regression technique) with an identity link with 95% CIs for the primary endpoint. Although, the participants will be randomly allocated to study groups, estimates will be adjusted for potential confounders if any significant differences are found between the study groups.

The intention-to-treat analysis will be applied to the per-protocol population. Robust standard errors will be estimated to account for clustering effects and possible correlations among participants receiving services from the same health facilities or exposure from same FHE.

LISREL version 8.8, SPSS version 23.0 and/or Stata version 14.1 will be used for analysis.

### Analysis of psychometric data

Psychometric data will be first subjected to a Confirmatory Factor Analysis (CFA) to confirm the measurement validity of the constructs. Scales for the constructs will be refined if the model produces a low goodness of fit. Subsequently, reliability of the final scales will be tested through Ordinal Alpha, Coefficient H, and Composite Reliability, while CFA will establish the validity of the scales. Once the scales’ psychometric properties are confirmed to be reliable and valid, we will the validate the final behavioural model for post-partum uptake of modern contraception through Structural Equation Modelling (SEM).

### Data monitoring

The principal investigators and research team will closely supervise activities throughout the intervention and study period. Data collection tools will be translated under the close supervision of the principal investigator for accuracy. To ensure data quality, the principal investigator will conduct trainings for the enumerators. Additionally, the PI and study coordinator will regularly monitor adherence to study protocol and data collection processes.

During the study period, the research team will re-interview between two and 3 % of participants with shortened questionnaires at both time points. Intra-variability of interviewers will be tested by comparing data collected by the research team and the enumerators. If inconsistencies are noted, the research team will conduct a refresher training for enumerators. Finally, use of dual data entry will assure accurate entry and identify data entry operators who are routinely inconsistent.

### Harms (Adverse Events)

#### Risks to participants

As family planning is considered a taboo in Pakistan women often receive contraceptive services without informing other household members, making loss of confidentiality a potential risk for study participants. Conducting baseline and follow-up interviews at clients’ homes may, therefore, lead to a loss of confidentiality. To mitigate this risk, enumerators will take consent from participants to conduct household-based interviews at the time of enrolment. Further, participants will have the option to select another, more private location to complete the interviews. Participants will have time to discuss participation with the heads of the household (mother-in-law or husband) prior to enrolment.

The use of mHealth approaches further increases a possible breach of confidentiality as it is possible for a husband or other family member to answer a phone call, hear a voice message or see a text message and provoke negative reactions. To prevent this, enumerators will ask participants a series of questions including whether it is acceptable to contact them on their mobile phone (via message or call), who owns the mobile phone, if a certain time during the day is more suitable to receive messages or calls, and whether their husband is aware of their use of or interest in family planning methods. It is pertinent to note that voice messages and interactive phone calls will be completed solely by females. Additionally, the organization’s name will be reflected in the voice and text messages to avoid any ambiguities that may occur from anonymity. Lastly, in the case of the interactive phone calls, if the call is answered by someone other than the participant – the health worker will discuss issues related to general health.

The most recent DHS report (12, 13) revealed that the most common form of violence in this context is husbands’ desire to know the whereabouts of their wife and jealousy if they talk to other men. This study addresses these by completing interviews in the household setting and ensuring females complete all voice messages and phone calls. Though chances are minimal, in the case of intimate partner violence (IPV) participants will be connected to local organisations that provide IPV services such as PANAH Shelter and Madadgar. During recruitment, enumerators will ask participants to contact MSS (i.e. helpline, FHE, project team) in the case of IPV to seek help and/or to discontinue the study. A member of the MSS Health Services Department (HSD) will be nominated to document and track any reports of IPV amongst participants and report this to the project staff along with the appropriate actions to take.

Study participants may experience side effects from the contraceptive methods. This, however, is a risk due to contraceptive use and not due to their participation in the study. Study participants experiencing severe complications will be connected to a nearby MSS service provider for follow-up care.

Lastly, participants may choose to stop receiving messages or phone calls from the counsellor at any point during the intervention. They may contact the field health educator, the MSS helpline, respond to a text message to cancel subscription or contact the research coordinator to discontinue participation. Participants will receive monthly reminders on how to opt-out of the intervention.

#### Risks to enumerators

The main risk to enumerators is related to safety concerns during household visits. The following measures will be in place to mitigate these risks: (1) study sites will consist of areas with on-going MSS operations; (2) study sites will be areas where MSS has strong relations with the community stakeholders; (3) all enumerators will be local and belong to the district where they are working; all enumerators will have previous experience conducting field-based surveys; (4) if necessary, FHEs will guide enumerators for locating participants’ households; (5) enumerators will travel using public transport which is considered safe in these areas; (6) senior field supervisors, who oversee between four and five Suraj SFs, will be engaged for support as needed; and (7) MSS has a separate security department to ensure safety of its employees.

#### Confidentiality

Several measures will be taken to protect the confidentiality and anonymity of study participants. Interviews will be conducted in complete privacy at participants’ homes and no personal identifiers will be recorded on the main questionnaire. Participants’ contact information and names will be stored separately on contact informant ion sheets, which will link to the questionnaire data using a unique personal ID for each participant. These contact sheets will be stored separately from the questionnaires in a locked cabinet under the custody of a senior research team member in the field. All data forms and questionnaires will be under the custody of an authorized research personnel in locked cabinets. Moreover, data entry software, accessible only to data entry clerks, data managers and study investigators, and the full database, restricted to the data manager and study investigators, will be password protected. Study participants will not be identified by name in any report or publication from this study. Loss of confidentiality may occur if these procedures are inadvertently breached, however, the research team will make all possible efforts to prevent this.

#### Access to data

Only research team members in the field will have access to field level data including the filled questionnaires and participant contact sheets. The data entry software will be password protected and accessible to data entry clerks, data managers and study investigators while the final complete database will be password protected and only accessible to data managers and study investigators.

#### Ancillary / Post-trial care

If either intervention proves to improve health outcomes all participants will have the opportunity to receive health education message through the mHealth approach following the intervention period.

Additionally, following the intervention participants will be able to contact FHEs, the MSS helpline, or visit a Suraj SF to receive additional counseling, health education, support for managing family planning side effects and family planning services.

#### Dissemination policy

Findings from PPFP will be shared with various stakeholders including donors, technical partners, local government, and NGOs through briefs, reports, and national level seminars. Additionally, the research team aims to disseminate findings through relevant international and regional conferences and to publish results from the study in a peer-reviewed international journal.

## Discussion

The study findings will highlight the challenges and best practices in proving quality PPFP services. Primarily, the study findings will be used to improve effectiveness of the program in promoting uptake of quality PPFP services and long-term method continuation. In addition, the study findings will help MSS amend for or include context specific information (social and cultural aspects) in the training curriculum and develop strategies for counselling, supervision and support mechanisms to ensure that providers and field health educators are able to meet the needs of PPFP clients in the community with quality information and services. The study findings will serve as a strong reference for the use of mobile technology in promoting health behaviours especially pertaining to reproductive health and family planning. The findings will also have strong relevance for public sector programs such as LHWs who are responsible to pregnant women with birth preparedness and ensuring essential postnatal care is adopted by women. Moreover, the research evidence will help develop effective evidence-based strategies to further increase awareness of, demand for and use of family planning and healthy timing and spacing of pregnancies during the postpartum period.

## Additional files

Additional file 1: Baseline Questionnaire for the Study (English). (PDF 435 kb)

Additional file 2: Follow-up Questionnaire for the Study (English). (PDF 487 kb)

Additional file 3: Baseline Questionnaire for the Study (Urdu). (PDF 7650 kb)

Additional file 4: Follow-up Questionnaire for the Study (Urdu). (PDF 5350 kb)

## Data Availability

Not Applicable.

## Abbreviations

CFA: Confirmatory Factor Analysis
CI: Confidence Interval
DHS: Demographic and Health Survey
FHE: Field Health Educator
FP: Family Planning
HSD: Health Services Department
IPV: Intimate Partner Violence
IUCD: Intra-uterine Contraceptive Device
LHW: Lady Health Worker
MSS: Marie Stopes Society
MWRA: Married Women of Reproductive Age
NBC: National Bioethics Committee
PI: Principal Investigator
PPFP: Post-partum Family Planning
SF: Social Franchise
TFR: Total Fertility Rate
WHO: World Health Organisation
