# Supplementary material for: A study protocol for an mHealth, multi-centre randomized control trial to promote use of postpartum contraception amongst rural women in Punjab, Pakistan
Source: BMC Pregnancy Childbirth. 2019 Aug 8;19:283. doi: 10.1186/s12884-019-2427-z (PMC6686472; doi:10.1186/s12884-019-2427-z)
Supplement: Supplementary file 1 — Baseline Questionnaire for the Study (English). (PDF 435 kb) [file 12884_2019_2427_MOESM1_ESM.pdf]

Baseline questionnaire:

SECTION 1: SOCIO-DEMOGRAPHIC CHARACTERISTICS			
	Record the current time	Hours [ ] Minutes [ ]	
First, I would like to ask some information about you and your husband			
**Q.no**	**Questions and Filters**	**Coding categories**	**Skip Pattern**
101	What is your approximate age in years?	Age in years [ ]	
102	What is your husband's approximate age in years?	Age in years [ ]	
103	What was your approximate age at the time of your marriage?	Age in years [ ]	
104	What is your highest level of education completed?	Illiterate 1<br>Can read, write and perform simple sums 2<br>Primary (1 to 5) 3<br>Middle (6 to 8) 4<br>Secondary (9 to 10) 5<br>Intermediate (11 to 12) 6<br>Graduate/Postgraduate (13+) 7	
105	What is the highest level of education completed by your husband?	Illiterate 1<br>Can read, write and perform simple sums 2<br>Primary (1 to 5) 3<br>Middle (6 to 8) 4<br>Secondary (9 to 10) 5<br>Intermediate (11 to 12) 6<br>Graduate/Postgraduate (13+) 7	
106	What is your occupation, that is, what kind of work do you mainly do, to earn income?	Housewife 1<br>Agriculture/farming 2<br>Shopkeeper 3<br>Tailor 4<br>Retired 5<br>Student 6<br>Others<br>specify_______________________	
107	What is your husband occupation, that is, what kind of work does he mainly do, to earn income?	Unemployed 1<br>Agriculture/farming 2<br>Shopkeeper 3<br>Tailor 4<br>Retired 5<br>Student 6<br>Others<br>specify_______________________	
108	Do you live in a joint family (with in-laws) or separate?	Joint 1<br>Nuclear 2	
**SECTION 2: REPRODUCTION**			
**Now I would like to ask about your reproductive history**			
201	How many times have you been pregnant? (including current pregnancy)	Number of pregnancies [ ]	If '1' ask 205
202	Have you ever had a pregnancy that miscarried, was aborted, or ended in a stillbirth?	Yes 1<br>No 2	→ ask 204

203	How many pregnancies have you had that miscarried, was aborted, or ended in a stillbirth?	Number ☐	
204	How many children do you currently have? (Please, specify the number of living children)	Boy ☐ Girls ☐	
205	When you got pregnant, did you want to become pregnant then, did you want to wait until later, or did you not want to have any (more) children at all?	Then 1<br>Later 2<br>Not at all 3	
206	After the birth of the child you are expecting now, how long would you like to wait before the birth of another child?	Years ☐☐<br>Months ☐☐<br>No more children 98	
207	After the birth of the child you are expecting now, how long would your husband like to wait before the birth of another child?	Years ☐☐<br>Months ☐☐<br>No more children 98	
208	**[ask if total pregnancies in Q122 > 1, otherwise ask Q130]**<br>What was the outcome of your last pregnancy?	Live birth 1<br>Still birth 2<br>Miscarriage 3<br>Aborted 4	
209	**[ask if total pregnancies in Q122 > 1, otherwise ask Q130]**<br>When was your last pregnancy ended?	Day ☐☐<br>Month ☐☐<br>Year ☐☐☐☐	
210	**[Check Q125: ask if participant currently have at least one alive child, otherwise ask Q133]**<br>What is the approximate age of your youngest child?	Years ☐☐<br>Months ☐☐	
211	**[ask if total children in Q125 > 1, otherwise ask Q133. Ask the name of child who age is reported in Q210]**<br>What is the age of your elder to [Name Q210 child]?	Years ☐☐<br>Months ☐☐	
**SECTION 3: KNOWLEDGE OF MATERNAL AND NEWBORN HEALTH**			
Now I would like to ask some question to know what kind of care is required during antenatal, childbirth and post-delivery for mother and newborn			
301	What key things can you do to prepare for the birth of a child?<br><br>**[MULTIPLE RESPONSES ARE ALLOWED]**	Pre-identify the health centre 1<br>Pre-identify the skilled birth attendant 2<br>Make prior arrangements of transport for taking pregnant woman to health centre 3<br>Save money to arrange transport and to bear the expenses of delivery 4<br>Arrange blood donation in case of any emergency during labor/birth 5<br>Have antenatal checkups 6<br>**Others (specify)**<br>a) ____________________<br>b) ____________________<br>c) ____________________<br>Don't know 98	

302	What are the danger signs that may occur during pregnancy and require immediate care from skilled healthcare provider?<br><br>**PROBE: ANYTHING ELSE?**<br><br>**[MULTIPLE RESPONSES ARE ALLOWED]**	Breathing difficulty 1<br>Vaginal bleeding 2<br>Convulsions or loss of consciousness / severe headache / blurred vision 3<br>Severe abdominal pain 4<br>Foul-smelling or meconium-stained discharge/fluid from vagina 5<br>Decreased or absent fetal movements 6<br>**Others (specify)**<br>a) ________________<br>b) ________________<br>c) ________________<br>Don't know 98	
303	What is the minimum number of antenatal check-ups recommended during pregnancy?	Number [ ][ ]<br>Don't know 98	
304	When should they start?	1st trimester 1<br>2nd trimester 2<br>3rd trimester 3<br>Don't know 98	
305	How many Tetanus Toxoid injections required during pregnancy?	Number [ ][ ]<br>Don't know 98	
306	What are the danger signs of mother that may occur during postpartum period (within 42 days after delivery) and require immediate care from skilled healthcare provider?<br><br>**PROBE: ANYTHING ELSE?**<br>**[MULTIPLE RESPONSES ARE ALLOWED]**	Heavy vaginal bleeding 1<br>Breathing difficulty 2<br>Fever 3<br>Abdominal pain 4<br>Severe headache/blurred vision 5<br>Convulsions or loss of consciousness 6<br>Foul smelling discharge 7<br>Behavior that indicates that she may hurt herself or her baby 8<br>**Others (specify)**<br>a) ________________<br>b) ________________<br>c) ________________<br>Don't know 98	
307	What is the minimum number of postnatal checkups recommended?	Number [ ][ ]<br>Don't know 98	
308	What are the danger signs of newborn that may occur during postpartum period (within 42 days after delivery) and require immediate care from skilled healthcare provider?<br><br>**PROBE: ANYTHING ELSE?**<br><br>**[MULTIPLE RESPONSES ARE ALLOWED]**	Breathing difficulty 1<br>Convulsions, spasms, or loss of consciousness 2<br>Cyanosis (blueness of the skin and mucous membranes) 3<br>Floppiness 4<br>Lethargy 5<br>Hot to touch (fever) 6<br>Cold to touch 7<br>Bleeding 8<br>Severe jaundice (yellowness of the skin and eyes) 9<br>Not feeding or sucking poorly 10<br>Diarrhea 11<br>Persistent vomiting or abdominal distention 12<br>Pus or redness of the umbilicus (navel), eyes, or skin 13<br>Swollen limb or joint 14<br>**Others (specify)**	

		a) ____________________<br>b) ____________________<br>c) ____________________<br>Don't know 98	
309	What should be done to keep the newborn warm immediately after the delivery?	Put the newborn in warm cloths 1<br>Skin-to-skin care 2<br>Don't know 98<br>Others specify ____________	
310	When should you start breastfeeding a new born?	Minutes [ ]<br>Hours [ ]<br>Days [ ]<br>Immediately 0<br>Don't know 98	
311	Till what age should a child be exclusively breast fed?	Age in months [ ]<br>Don't know 98	
312	When should the first vaccine be given to the baby?	Minutes [ ]<br>Hours [ ]<br>Days [ ]<br>At birth 0<br>Don't know 98	
**SECTION 4: ANTENATAL, DELIVERY, AND POST NATAL CARE**			
Now I would like to ask you about the care you received when you were pregnant last time, during last delivery, and during postpartum period i.e. 42 after the last birth			
401	Did you receive antenatal care when you were pregnant last time?	Yes 1<br>No 2	→ ask 405
402	How many antenatal care visits were made?	Number of visits [ ][ ]<br>Don't know 98	
403	Where did you receive antenatal care?	Your home 1<br>Other home 2<br>Government Hospital 3<br>Rural Health Centre (RHC) 4<br>Basic Health Unit (BHU) 5<br>Dispensary 6<br>Maternal Child Health (MCH) 7<br>Family Welfare Centre 8<br>Private hospital/clinic 9<br>Homeopath clinic 10<br>MSS Suraj SF 11<br>Others (specify)____________	
404	Who examined you for the antenatal care?	Doctor 1<br>Nurse 2<br>Midwife 3<br>Lady health visitor 4<br>Lady health worker 5<br>Hakim/Homeopath 6<br>Dispenser/Compounder 7<br>Dai/Traditional Birth Attendant 8<br>Others (specify)____________	

405	Where was the last child delivered?	Your home 1<br>Other home 2<br>Government Hospital 3<br>Rural Health Centre (RHC) 4<br>Basic Health Unit (BHU) 5<br>Maternal Child Health (MCH) 6<br>Private hospital/clinic 7<br>Homeopath clinic 8<br>MSS Suraj SF 9<br>Others (specify)___________________________	
406	Was (NAME) delivered by caesarean, that is, did they cut your belly open to take the baby out?	Yes 1<br>No 2	
407	Who assisted with the last delivery of your child?	No one 1<br>Doctor 2<br>Nurse 3<br>Midwife 4<br>Lady Health Visitor (LHV) 5<br>Dai/Traditional Birth Attendant (TBA) 6<br>Family Welfare Worker (FWW) 7<br>Hakim 8<br>Others (specify)___________________________	
408	Did you use any birth spacing method within 42 days after your last delivery?	Yes 1<br>No 2	→ ask 501
409	Which method were you / your husband adopted?	IUD 1<br>Implant 2<br>Injections 3<br>Pills 4<br>Condom 5<br>Periodic Abstinence 6<br>Withdrawal 7<br>Others (specify)___________________________	

SECTION 5: CONTRACEPTION

Now I would like to ask about your contraceptive knowledge and history

	**Methods**	**Have you ever heard of [method]?**	**Do you know where a person could go to get [method]?**	
501	a) Pills	Yes 1<br>No↓ 2	Yes 1<br>No 2	
502	b) IUD	Yes 1<br>No↓ 2	Yes 1<br>No 2	
503	c) Injection	Yes 1<br>No↓ 2	Yes 1<br>No 2	
504	d) Implant	Yes 1<br>No↓ 2	Yes 1<br>No 2	
505	e) Condom	Yes 1<br>No↓ 2	Yes 1<br>No 2	
506	f) Female sterilization	Yes 1<br>No↓ 2	Yes 1<br>No 2	
507	g) Male sterilization	Yes 1<br>No↓ 2	Yes 1<br>No 2	
508	h) Periodic abstinence	Yes 1<br>No↓ 2	Yes 1<br>No 2	
509	i) Withdrawal	Yes 1<br>No↓ 2	Yes 1<br>No 2	

510	j) Lactational Amenorrhea	Yes 1<br>No↓ 2	Yes 1<br>No 2	
511	k) Emergency contraception	Yes 1<br>No↓ 2	Yes 1<br>No 2	
512	l) Others	Yes 1<br>No↓ 2	Yes 1<br>No 2	
513	Have you <u>ever</u> used anything or tried in any way to delay or avoid getting pregnant?	Yes 1<br>No 2		→ ask 515
514	What is the main reason that you have never used any contraceptive method?	Want more children 1<br>Infrequent sex/No sex 2<br>Menopausal/Hysterectomy 3<br>Infertile/can't get pregnant 4<br>No menstruation after birth 5<br>Breastfeeding 6<br>Up to God, can't control 7<br>Opposed to family planning 8<br>Husband opposed 9<br>Other people opposed 10<br>Religious reasons 11<br>Knows no method 12<br>Knows no source 13<br>Health concerns 14<br>Fear of Side Effects 15<br>Lack of access/Too far 16<br>Cost too much 17<br>Inconvenient to use 18<br>Others (specify)____________ _____________		
Now I would like to ask about future intention to contraceptive method use				
515	Do you intend to use a contraceptive method to delay or avoid your next pregnancy after childbirth?	Yes 1<br>No 2<br>Undecided 3		→ ask 517
516	What is the main reason that you think you will not use a contraceptive method?	Infrequent sex/no sex 1<br>Breastfeeding 2<br>Up to god, can't control 3<br>Want more children 4<br>Respondent opposed 5<br>Husband opposed 6<br>Others opposed 7<br>Against religion 8<br>Knows no method 9<br>Knows no source 10<br>Health concerns 11<br>Fear of side effects 12<br>Lack of access/too far 13<br>Costs too much 14<br>Inconvenient to use 15<br>Others (specify)_____________________<br>Don't know/Can't say anything 98		
Now I would like to ask some questions to gauge your perceptions regarding contraception.				
	**INTENTION**			
517	Do you expect to adopt a modern FP method to space births?	Completely No 1<br>Somewhat No 2<br>Neither Yes nor No 3<br>Somewhat Yes 4<br>Completely Yes 5		

518	Do you want to adopt a modern FP `method to space births?	Completely No 1<br>Somewhat No 2<br>Neither Yes nor No 3<br>Somewhat Yes 4<br>Completely Yes 5	
519	Do you intend to adopt a modern FP method to space births?	Completely No 1<br>Somewhat No 2<br>Neither Yes nor No 3<br>Somewhat Yes 4<br>Completely Yes 5	
	**POSITIVE ATTITUDE**		
520	Do you think that using a modern FP method for child spacing is a useful thing?	Completely No 1<br>Somewhat No 2<br>Neither Yes nor No 3<br>Somewhat Yes 4<br>Completely Yes 5	
521	Do you think that using a modern FP method for child spacing is a wise thing to do?	Completely No 1<br>Somewhat No 2<br>Neither Yes nor No 3<br>Somewhat Yes 4<br>Completely Yes 5	
522	Do you think that using a modern FP method for child spacing is a beneficial thing to do?	Completely No 1<br>Somewhat No 2<br>Neither Yes nor No 3<br>Somewhat Yes 4<br>Completely Yes 5	
523	If there is no gap between children naturally, a modern FP method is in-effective in for spacing births?	Completely No 1<br>Somewhat No 2<br>Neither Yes nor No 3<br>Somewhat Yes 4<br>Completely Yes 5	
	**MYTHS AND MISCONCEPTIONS**		
524	Do you think using modern FP method for child spacing will make you a little ill?	Completely Yes 1<br>Somewhat Yes 2<br>Neither Yes nor No 3<br>Somewhat No 4<br>Completely No 5	
525	Do you think that you can become severely ill by using a modern FP method for child spacing?	Completely Yes 1<br>Somewhat Yes 2<br>Neither Yes nor No 3<br>Somewhat No 4<br>Completely No 5	
526	Do you think that using a modern FP method for child spacing is harmful for health?	Completely Yes 1<br>Somewhat Yes 2<br>Neither Yes nor No 3<br>Somewhat No 4<br>Completely No 5	
527	If you get some illness from using a modern FP method for child spacing, do you think that you will have to spend a lot of money on its treatment?	Completely Yes 1<br>Somewhat Yes 2<br>Neither Yes nor No 3<br>Somewhat No 4<br>Completely No 5	

	**PERCEIVED NORMS**		
528	Do the people who are close to you think that using modern FP method to space births is a good thing?	Completely No 1<br>Somewhat No 2<br>Neither Yes nor No 3<br>Somewhat Yes 4<br>Completely Yes 5	
529	Do people your area support modern FP methods for birth spacing?	Completely No 1<br>Somewhat No 2<br>Neither Yes nor No 3<br>Somewhat Yes 4<br>Completely Yes 5	
530	Do the people who are close to you think that a woman who uses a modern FP method to space births is wise?	Completely No 1<br>Somewhat No 2<br>Neither Yes nor No 3<br>Somewhat Yes 4<br>Completely Yes 5	
531	Do the people who are close to you/known to you think that you should use modern FP method to space the birth of children?	Completely No 1<br>Somewhat No 2<br>Neither Yes nor No 3<br>Somewhat Yes 4<br>Completely Yes 5	
	**PERSONAL AGENCY**		
532	Is the decision to adopt a modern family planning method to space births in your control?	Completely No 1<br>Somewhat No 2<br>Neither Yes nor No 3<br>Somewhat Yes 4<br>Completely Yes 5	
533	If you wanted to use a modern family planning method to space births, would it be difficult for you to do so?	Completely Yes 1<br>Somewhat Yes 2<br>Neither Yes nor No 3<br>Somewhat No 4<br>Completely No 5	
534	Is using modern methods of family planning to space birth easy for you?	Completely No 1<br>Somewhat No 2<br>Neither Yes nor No 3<br>Somewhat Yes 4<br>Completely Yes 5	

SECTION 6: HOUSEHOLD DECISION MAKING						
Now I am going to ask you about general decision making in your household. For each of the following situations, tell me who makes the final decision - yourself alone, your husband alone, by your mother-in-law alone or father-in-law alone, or any other person. You can name also more than one person if the decision is taken collectively						

Description	Self (respondent)	Husband	Mother-in-law	Father-in-law	Others
a. small household expenditures like toothpaste, soap, crockery etc.	☐	☐	☐	☐	☐
b. purchase of major household expenditures like TV, refrigerator, etc.	☐	☐	☐	☐	☐
c. female expenditures like clothes and jewellery	☐	☐	☐	☐	☐
d. your employment outside the home	☐	☐	☐	☐	☐
e. buying or selling property	☐	☐	☐	☐	☐
f. children's clothes	☐	☐	☐	☐	☐
g. place where to take the child in the case of illness	☐	☐	☐	☐	☐
h. place where you go yourself for medical help in the case of illness	☐	☐	☐	☐	☐
i. purchase of medicine	☐	☐	☐	☐	☐
j. children's education	☐	☐	☐	☐	☐
k. visiting relatives (when or where)	☐	☐	☐	☐	☐
l. number of children	☐	☐	☐	☐	☐
m. use of family planning method	☐	☐	☐	☐	☐

602	Do you go out of your house alone whenever you need medical help?	Yes 1<br>No 2	→ ask 701
603	Who usually accompanies you when you go out of you house to seek medical help?	Go alone 1<br>Accompanied by husband 2<br>Accompanied by mother-in-law 3<br>Others (specify)_____________________ 99	

SECTION 7: POVERTY ASSESSMENT			
**Read to respondent**: "I would like to ask you some questions about your living conditions. I realize some of these questions seem unrelated to healthcare, but all of these questions help us to understand your living situation. Please answer as honestly as possible, as this will allow us to better serve the community.<br>*Read questions to respondent exactly as written. Do not read out the response options. Circle the number corresponding closest to the respondent's answer. All questions must be answered.*			
701	In what province does the household live?	Baluchistan 1<br>Khyber Pakhtunkhwa 2<br>Sindh 3<br>Punjab 4	
702	How many household members are 13 years old or younger?	Five or more 1<br>Four 2<br>Three 3<br>Two 4<br>One 5<br>None 6	
703	How many children ages 5 to 13 who attend school?	Not all children/some children 1<br>All children, or no children ages 5 to 13 years 2	
704	How many household members work in elementary occupations (not senior officials, managers, professionals, technicians or associated professionals, clerks, salespeople, service or shop workers, skilled workers in agriculture or fishery, craft or trade workers, or plant/machinery operators)?	Two or more 1<br>One 2<br>None 3	

705	What is the highest educational level completed by the female head/spouse?	Less than Class 1 or no data 1<br>No female head/spouse 2<br>Class 1 or higher 3	
706	What is the main source of drinking water for the household?	Others 1<br>Hand pump, covered/closed well, motorized pump/tube well, or piped water 2	
707	What type of toilet is used by your household?	None or other 1<br>Flush connected to pit/septic tank or open drain 2<br>Flush connected to public sewerage 3	
708	Does the household own a refrigerator or freezer?	No 1<br>Yes 2	
709	Does the household own a television?	No 1<br>Yes 2	
710	Does the household own a motorcycle, scooter, car or other vehicle?	No 1<br>Yes 2	
**SECTION 8: DOMESTIC VIOLENCE ASSESSMENT**			
**Read to respondent**: *"Now I would like to ask you questions about some other important aspects of a woman's life. I know that some of these questions are very personal. Let me assure you that your answers are completely confidential and will not be told to anyone, and no one else will know that you were asked these questions. You do not have to answer these questions if you do not want to; you can just tell me to skip to the next question.*<br>*Read questions to respondent exactly as written. Do not read out the response options. Circle the number corresponding closest to the respondent's answer. All questions must be answered.*			
801	Have you ever been emotionally or physically abused by your husband or someone close to you?	No 1<br>Yes 2	→ ask 805
802	Within the six months, have you been hit, slapped, kicked or otherwise physically harmed by someone?	No 1<br>Yes 2	→ ask 805
803	If yes, by whom?	Husband 1<br>In laws 2<br>Other 3	
804	How many times?		
805	Since you've been pregnant, were you hit, slapped, kicked, or otherwise physically harmed by someone?	No 1<br>Yes 2	→ ask 811
806	If yes, by whom?	Husband 1<br>In laws 2<br>Other 3	
807	How many times?		
808	Which part you were hit most on your body?<br><br>[Only one answer is allowed]	Head 1<br>Face 2<br>Chest/Breast 3<br>Stomach/Abdomen 4<br>Upper back 5<br>Buttock 6<br>Leg 7	
809	What instrument used for violence?	Using hand 1<br>Using instrument like knife/wood/pipe etc 2<br>Others (specify)____________________________	
810	What was the severity of* violence?	No / Yes<br>Injuries and/or lasting pain 1 2<br>Bruises, cuts and/or continuing pain 1 2<br>Severe contusions, burns, broken bones 1 2<br>Internal injury, permanent injury 1 2	

811	In the last six months, has your husband physically forced you to have sexual intercourse with him even when you did not want to?	No 1<br>Yes 2	→ ask 913
812	How many times?		
813	Are you afraid of your husband?	No 1<br>Yes 2	
	**THANK YOU FOR YOUR PARTICIPATION**		
