# Supplementary material for: A study protocol for an mHealth, multi-centre randomized control trial to promote use of postpartum contraception amongst rural women in Punjab, Pakistan
Source: BMC Pregnancy Childbirth. 2019 Aug 8;19:283. doi: 10.1186/s12884-019-2427-z (PMC6686472; doi:10.1186/s12884-019-2427-z)
Supplement: Supplementary file 2 — Follow-up Questionnaire for the Study (English). (PDF 487 kb) [file 12884_2019_2427_MOESM2_ESM.pdf]

SECTION 1: ANTENATAL, DELIVERY, AND POST NATAL CARE

Now I would like to ask you about the care you received when you were pregnant last time, during delivery, and during postpartum period i.e. 15 after last birth

	Record the current time	Hours [ ] Minutes [ ]	
**Q.no**	**Questions and Filters**	**Coding categories**	**Skip Pattern**
1	Did you receive antenatal care when you were pregnant last time?	Yes 1<br>No 2	 → ask Q5
2	How many antenatal care visits were made?	Number of visits [ ][ ]<br>Don't know 98	
3	Where did you receive antenatal care?<br><br>**(Name of place)**	**HOME**<br>Your home 1<br>Other home 2<br>**PUBLIC SECTOR**<br>Government Hospital 3<br>Rural Health Centre (RHC) 4<br>Basic Health Unit (BHU) 5<br>Dispensary 6<br>Maternal Child Health (MCH) 7<br>Family Welfare Centre 8<br>**PRIVATE/NGO MEDICAL SECTOR**<br>Private hospital/clinic 9<br>Homeopath clinic 10<br>Others (specify)_______________________	
4	Who examined you for the antenatal care?	**HEALTH PERSON**<br>Doctor 1<br>Nurse 2<br>Midwife 3<br>Lady health visitor 4<br>**OTHER PERSON**<br>Private hospital/clinic 5<br>Lady health worker 6<br>Hakim/Homeopath 7<br>Dispenser/Compounder 8<br>Dai/Traditional Birth Attendant 9<br>Others (specify)_______________________	
5	Where was the last child delivered?	**HOME**<br>Your home 1<br>Other home 2<br>**PUBLIC SECTOR**<br>Government Hospital 3<br>Rural Health Centre (RHC) 4<br>Basic Health Unit (BHU) 5<br>Maternal Child Health (MCH) 6<br>**PRIVATE/NGO MEDICAL SECTOR**<br>Private hospital/clinic 7<br>Homeopath clinic 8<br>MSS Suraj SF 9<br>Others (specify)_______________________	
6	What was the mode of last delivery?	Normal 1<br>Breech delivery 2<br>Instrumental delivery 3<br>Elective c-section 4<br>Emergency c-section 5<br>Vacuum applied 6<br>Others (specify)_______________________	

7	Who assisted with the last delivery of your child?	No one 1<br>**HEALTH PERSON**<br>Doctor 2<br>Nurse 3<br>Midwife 4<br>Lady Health Visitor (LHV) 5<br>Lady Health Worker (LHW) 6<br>Dai/Traditional Birth Attendant (TBA) 7<br>Family Welfare Worker (FWW) 8<br>Hakim 9<br>Relative/friends (not a dai) 10<br>Others (specify)________________________	
8	Did you use contraception after your last delivery	Yes 1<br>No 2	→ ask Q12
9	Which contraception did you use?	IUCD 1<br>Implant 2<br>Oral pills 3<br>Condom 4<br>Injection 5<br>Female sterilization 6<br>Periodic abstinence 7<br>Withdrawal 8<br>Other (specify)___________________________	
10	When did you uptake this contraception after your last delivery	Days ago _ _<br>Weeks ago _ _	
11	Where did you get this contraceptive	Government hospital/clinic 1<br>Rural health centre 2<br>Basic health unit 3<br>Maternity home 4<br>Private hospital/clinic 5<br>Suraj centre 6<br>Homeopathic clinic 7<br>Pharmacy/medical store 8<br>Husband knows 9<br>Other (specify)__________________________	
12	Why you did not use any contraception?	Want more children 1<br>Infrequent sex/No sex 2<br>Menopausal/Hysterectomy 3<br>Infertile/can't get pregnant 4<br>No menstruation after birth 5<br>Breastfeeding 6<br>Up to God, can't control 7<br>Opposed to family planning 8<br>Husband opposed 9<br>Other people opposed 10<br>Religious reasons 11<br>Knows no method 12<br>Knows no source 13<br>Health concerns 14<br>Fear of Side Effects 15<br>Lack of access/Too far 16<br>Cost too much 17<br>Inconvenient to use 18<br>Others (specify)_____________________ 19	

SECTION 2: KNOWLEDGE OF MATERNAL AND NEWBORN HEALTH			
Now I would like to ask some question to know what kind of care is required during antenatal, childbirth and post-delivery for mother and newborn			
13	What key things can you do to prepare for the birth of a child?<br><br>**[MULTIPLE RESPONSES ARE ALLOWED]**	Pre-identify the health centre 1<br>Pre-identify the skilled birth attendant 2<br>Make prior arrangements of transport for taking pregnant woman to health centre 3<br>Save money to arrange transport and to bear the expenses of delivery 4<br>Arrange blood donation in case of any emergency during labor/birth 5<br>Have antenatal checkups 6<br>**Others (specify)**<br>a) ______________________________<br>b) ______________________________<br>c) ______________________________<br>Don't know 98	
14	What are the danger signs that may occur during pregnancy and require immediate care from skilled healthcare provider?<br><br>**PROBE: ANYTHING ELSE?**<br><br>**[MULTIPLE RESPONSES ARE ALLOWED]**	Breathing difficulty 1<br>Vaginal bleeding 2<br>Convulsions or loss of consciousness / severe headache / blurred vision 3<br>Severe abdominal pain 4<br>Foul-smelling or meconium-stained discharge/fluid from vagina 5<br>Decreased or absent fetal movements 6<br>**Others (specify)**<br>a) ______________________________<br>b) ______________________________<br>c) ______________________________<br>Don't know 98	
15	What is the minimum number of antenatal checkups recommended during pregnancy?	Number [ ][ ]<br>Don't know 98	
16	When should they start?	1st trimester 1<br>2nd trimester 2<br>3rd trimester 3<br>Don't know 98	
17	How many Tetanus Toxoid injections required during pregnancy?	Number [ ][ ]<br>Don't know 98	
18	What are the danger signs of mother that may occur during postpartum period (within 42 days after delivery) and require immediate care from skilled healthcare provider?<br><br>**PROBE: ANYTHING ELSE?**<br>**[MULTIPLE RESPONSES ARE ALLOWED]**	Heavy vaginal bleeding 1<br>Breathing difficulty 2<br>Fever 3<br>Abdominal pain 4<br>Severe headache/blurred vision 5<br>Convulsions or loss of consciousness 6<br>Foul smelling discharge 7<br>Behavior that indicates that she may hurt herself or her baby 8<br>**Others (specify)**<br>a) ______________________________<br>b) ______________________________<br>c) ______________________________<br>Don't know 98	

19	What is the minimum number of postnatal checkups recommended?	Number [ ][ ]<br>Don't know 98	
20	What are the danger signs of newborn that may occur during postpartum period (within 42 days after delivery) and require immediate care from skilled healthcare provider?<br><br>**PROBE: ANYTHING ELSE?**<br><br>**[MULTIPLE RESPONSES ARE ALLOWED]**	Breathing difficulty 1<br>Convulsions, spasms, or loss of consciousness 2<br>Cyanosis (blueness of the skin and mucous membranes) 3<br>Floppiness 4<br>Lethargy 5<br>Hot to touch (fever) 6<br>Cold to touch 7<br>Bleeding 8<br>Severe jaundice (yellowness of the skin and eyes) 9<br>Not feeding or sucking poorly 10<br>Diarrhea 11<br>Persistent vomiting or abdominal distention 12<br>Pus or redness of the umbilicus (navel), eyes, or skin 13<br>Swollen limb or joint 14<br>**Others (specify)**<br>a) ____________________<br>b) ____________________<br>c) ____________________<br>Don't know 98	
21	What should be done to keep the newborn warm immediately after the delivery?	Put the newborn in warm cloths 1<br>Skin-to-skin care 2<br>Don't know 98<br>Others specify ____________________	
22	When should you start breastfeeding a new born?	Minutes [ ]<br>Hours [ ]<br>Days [ ]<br>Immediately 0<br>Don't know 98	
23	Till what age should a child be exclusively breast fed?	Age in months [ ]<br>Don't know 98	
24	When should the first vaccine be given to the baby?	Minutes [ ]<br>Hours [ ]<br>Days [ ]<br>At birth 0<br>Don't know 98	

SECTION 3: CONTRACEPTION

Now I would like to ask about the contraceptive knowledge and history

	Methods	Have you ever heard of [method]?	Do you know where a person could go to get [method]?	
25	a) Pills	Yes 1<br>No↓ 2	Yes 1<br>No 2	
26	b) IUD	Yes 1<br>No↓ 2	Yes 1<br>No 2	
27	c) Injection	Yes 1<br>No↓ 2	Yes 1<br>No 2	

28	d) Implant	Yes 1<br>No↓ 2	Yes 1<br>No 2	
29	e) Condom	Yes 1<br>No↓ 2	Yes 1<br>No 2	
30	f) Female sterilization	Yes 1<br>No↓ 2	Yes 1<br>No 2	
31	g) Male sterilization	Yes 1<br>No↓ 2	Yes 1<br>No 2	
32	h) Periodic abstinence	Yes 1<br>No↓ 2	Yes 1<br>No 2	
33	i) Withdrawal	Yes 1<br>No↓ 2	Yes 1<br>No 2	
34	j) LAM	Yes 1<br>No↓ 2	Yes 1<br>No 2	
35	k) Emergency contraception	Yes 1<br>No↓ 2	Yes 1<br>No 2	
36	l) Others	Yes 1<br>No↓ 2	Yes 1<br>No 2	

37	Have you ever used or tried anything to delay or avoid getting pregnant?	Yes 1<br>No 2	→ ask Q39
38	If no, what is the main reason that you did not try any contraceptive method?	Want more children 1<br>Infrequent sex/No sex 2<br>Menopausal/Hysterectomy 3<br>Infertile/can't get pregnant 4<br>No menstruation after birth 5<br>Breastfeeding 6<br>Up to God, can't control 7<br>Opposed to family planning 8<br>Husband opposed 9<br>Other people opposed 10<br>Religious reasons 11<br>Knows no method 12<br>Knows no source 13<br>Health concerns 14<br>Fear of Side Effects 15<br>Lack of access/Too far 16<br>Cost too much 17<br>Inconvenient to use 18<br>Interferes with body's normal processes 19<br>Others (specify)______________________<br>Don't know .98	
Now I would like to ask about future intention to contraceptive method use			
39	**[ask if participant is not currently using any contraceptive method, otherwise ask Q154]**<br>Do you intend to use a contraceptive method to delay or avoid your next pregnancy after childbirth?	Yes 1<br>No 2	→ ask Q41

40	What is the main reason that you think you will not use a contraceptive method?	Infrequent sex/no sex 1<br>Breastfeeding 2<br>Up to god, can't control 3<br>Want more children 4<br>Respondent opposed 5<br>Husband opposed 6<br>Others opposed 7<br>Against religion 8<br>Knows no method 9<br>Knows no source 10<br>Health concerns 11<br>Fear of side effects 12<br>Lack of access/too far 13<br>Costs too much 14<br>Inconvenient to use 15<br>Others (specify)____________________<br>Don't know/Can't say anything 98	
**[ask Q151 to Q153 if participant is not currently using any contraceptive method, otherwise ask Q154]**<br>Now I would like to ask some questions to gauge your perceptions regarding contraception.			
	**INTENTION**		
41	Do you expect to adopt a modern FP method to space births?	Completely No 1<br>Somewhat No 2<br>Neither Yes nor No 3<br>Somewhat Yes 4<br>Completely Yes 5	
42	Do you want to adopt a modern FP `method to space births?	Completely No 1<br>Somewhat No 2<br>Neither Yes nor No 3<br>Somewhat Yes 4<br>Completely Yes 5	
43	Do you intend to adopt a modern FP method to space births?	Completely No 1<br>Somewhat No 2<br>Neither Yes nor No 3<br>Somewhat Yes 4<br>Completely Yes 5	
	**POSITIVE ATTITUDE**		
44	Do you think that using a modern FP method for child spacing is a useful thing?	Completely No 1<br>Somewhat No 2<br>Neither Yes nor No 3<br>Somewhat Yes 4<br>Completely Yes 5	
45	Do you think that using a modern FP method for child spacing is a wise thing to do?	Completely No 1<br>Somewhat No 2<br>Neither Yes nor No 3<br>Somewhat Yes 4<br>Completely Yes 5	
46	Do you think that using a modern FP method for child spacing is a beneficial thing to do?	Completely No 1<br>Somewhat No 2<br>Neither Yes nor No 3<br>Somewhat Yes 4<br>Completely Yes 5	
47	If there is no gap between children naturally, a modern FP method is in effective in for spacing births?	Completely No 1<br>Somewhat No 2<br>Neither Yes nor No 3<br>Somewhat Yes 4<br>Completely Yes 5	

	**MYTHS AND MISCONCEPTIONS**		
48	Do you think using modern FP method for child spacing will make you a little ill?	Completely Yes 1<br>Somewhat Yes 2<br>Neither Yes nor No 3<br>Somewhat No 4<br>Completely No 5	
49	Do you think that you can become severely ill by using a modern FP method for child spacing?	Completely Yes 1<br>Somewhat Yes 2<br>Neither Yes nor No 3<br>Somewhat No 4<br>Completely No 5	
50	Do you think that using a modern FP method for child spacing is harmful for health?	Completely Yes 1<br>Somewhat Yes 2<br>Neither Yes nor No 3<br>Somewhat No 4<br>Completely No 5	
51	If you get some illness from using a modern FP method for child spacing, do you think that you will have to spend a lot of money on its treatment?	Completely Yes 1<br>Somewhat Yes 2<br>Neither Yes nor No 3<br>Somewhat No 4<br>Completely No 5	
	**PERCEIVED NORMS**		
52	Do the people who are close to you think that using modern FP method to space births is a good thing?	Completely No 1<br>Somewhat No 2<br>Neither Yes nor No 3<br>Somewhat Yes 4<br>Completely Yes 5	
53	Do people your area support modern FP methods for birth spacing?	Completely No 1<br>Somewhat No 2<br>Neither Yes nor No 3<br>Somewhat Yes 4<br>Completely Yes 5	
54	Do the people who are close to you think that a woman who uses a modern FP method to space births is wise?	Completely No 1<br>Somewhat No 2<br>Neither Yes nor No 3<br>Somewhat Yes 4<br>Completely Yes 5	
55	Do the people who are close to you/known to you think that you should use modern FP method to space the birth of children?	Completely No 1<br>Somewhat No 2<br>Neither Yes nor No 3<br>Somewhat Yes 4<br>Completely Yes 5	
	**PERSONAL AGENCY**		
56	Is the decision to adopt a modern family planning method to space births in your control?	Completely No 1<br>Somewhat No 2<br>Neither Yes nor No 3<br>Somewhat Yes 4<br>Completely Yes 5	
57	If you wanted to use a modern family planning method to space births, would it be difficult for you to do so?	Completely Yes 1<br>Somewhat Yes 2<br>Neither Yes nor No 3<br>Somewhat No 4<br>Completely No 5	
58	Is using modern methods of family planning to space birth easy for you?	Completely No 1<br>Somewhat No 2<br>Neither Yes nor No 3<br>Somewhat Yes 4<br>Completely Yes 5	

SECTION 4: EVALUATION OF COUNSELING (VOICE MESSAGES AND SHORT MESSAGING SERVICE)			
Now I am going to ask you about voice messages that you have received during your pregnancy.			
59	Have you received any voice messages from MSS during your last pregnancy or within 42 days after the birth?	Yes 1<br>No 2	 → ask Q70
60	How many times have you received voice messages?	No. of calls/messages [ ][ ]<br>Don't remember 98	
61	What was the language of voice messages?	Urdu 1<br>English 2<br>Punjabi 3<br>Sindhi 4	
62	Was it easy to understand the voice messages?	Yes 1<br>No 2	
63	What was the average length or duration of voice messages?	Seconds [ ][ ]<br>Minutes [ ][ ]	
64	Did the MSS sent voice messages at a time that was convenient for you to listen?	Yes 1<br>No 2	 → ask Q66
65	If no, what should be the best time to send you the voice messages?	Time _ _ : _ _	
66	Were you comfortable with the following actions made by MSS?<br><br>Timing of voice messages<br>Duration of voice messages<br>Language of voice messages<br>Number of voice messages	**Yes** **No**<br>1 2<br>1 2<br>1 2<br>1 2	
67	What kind of information was provided by MSS to you via voice messages?<br><br>**[MULTIPLE RESPONSES ALLOWED]**	Minimum no. of antenatal care visits 1<br>Timing of antenatal care visits 2<br>How many tetanus toxoid injections required during pregnancy 3<br>Provide information about nutrition supplementary during pregnancy 4<br>Highlighted danger signs of pregnancy 5<br>What to do if any danger signs of pregnancy appeared 6<br>Where to go in case of emergency during pregnancy 7<br>How and what to plan for delivery 8<br>Arrangement of necessary things (money, blood donor, travel, pre-identify skilled provider and health facility) for delivery 9<br>Provided information about breastfeeding (When should start, till what age) 10<br>Counseled on child immunization 11<br>Highlighted newborn danger signs 12<br>Number of immunization should a child receive 13<br>Highlighted essential newborn care practices 14<br>Highlighted importance of post-partum family planning/birth spacing 15	

		Place where to obtain FP method after delivery 16<br>Minimum no. of postnatal care visits 17<br>Timing of postnatal care visits 18<br>Manage/identify postnatal complications 19<br>Counseled on danger signs after delivery 20<br>**Others (specify)**<br>d) ________________________<br>e) ________________________<br>f) ________________________<br>g) ________________________	
68	Are you satisfied with the information/counselling provided by the MSS via voice messages?	Yes – Completely satisfied 1<br>Yes – Somewhat satisfied 2<br>Neutral/Just fine 3<br>No – Somewhat unsatisfied 4<br>No – completely unsatisfied 5	
69	How useful/effective was the information/counselling provided by MSS via voice messages?	Completely useful/effective 1<br>Somewhat useful/effective 2<br>Neutral/Just fine 3<br>Somewhat useless/ineffective 4<br>Completely useless/ineffective 5	
Now, I would like to ask some questions about Short Messaging Service (SMS)			
70	Have you received any short messages from MSS during your last pregnancy or within 42 days after the birth?	Yes 1<br>No 2	→ ask Q80
71	On average, how many SMS have you received?	No. of SMS [ ][ ]<br>Don't remember 98	
72	What was the language of short messages?	Urdu 1<br>Roman Urdu 2<br>English 3<br>Punjabi 4<br>Sindhi 5	
73	Was it easy to understand the message?	Yes 1<br>No 2	
74	Did MSS send the SMS at a time that was convenient for you to read?	Yes 1<br>No 2	→ ask Q76
75	If no, what should be the best time to send you the SMS?	Time _ _ : _ _	
76	Were you comfortable with the following actions made by MSS?<br><br>**Yes** **No**<br>Timing of SMS 1 2<br>Length of SMS 1 2<br>Language of SMS 1 2<br>Number of SMS recieved 1 2		

77	What kind of information was provided by MSS to you via SMS?<br><br>**[MULTIPLE RESPONSES ALLOWED]**	Minimum no. of antenatal care visits 1<br>Timing of antenatal care visits 2<br>How many tetanus toxoid injections required during pregnancy 3<br>Provide information about nutrition supplementary during pregnancy 4<br>Highlighted danger signs of pregnancy 5<br>What to do if any danger signs of pregnancy appeared 6<br>Where to go in case of emergency during pregnancy 7<br>How and what to plan for delivery 8<br>Arrangement of necessary things (money, blood donor, travel, pre-identify skilled provider and health facility) for delivery 9<br>Provided information about breastfeeding (When should start, till what age) 10<br>Counseled on child immunization 11<br>Highlighted newborn danger signs 12<br>Number of immunization should a child receive 13<br>Highlighted essential newborn care practices 14<br>Highlighted importance of post-partum family planning/birth spacing 15<br>Place where to obtain FP method after delivery 16<br>Minimum no. of postnatal care visits 17<br>Timing of postnatal care visits 18<br>Manage/identify postnatal complications 19<br>Counseled on danger signs after delivery 20<br>**Others (specify)**<br>h) ________________________<br>i) ________________________<br>j) ________________________<br>k) ________________________	
78	Are you satisfied with the information/counselling provided by MSS via SMS?	Yes – Completely satisfied 1<br>Yes – Somewhat satisfied 2<br>Neutral/Just fine 3<br>No – Somewhat unsatisfied 4<br>No – completely unsatisfied 5	
79	How useful/effective was the information/counselling provided by MSS via SMS?	Completely useful/effective 1<br>Somewhat useful/effective 2<br>Neutral/Just fine 3<br>Somewhat useless/ineffective 4<br>Completely useless/ineffective 5	
Now, I would like to ask some questions about Phone Calls			
80	Have you received any phone call from MSS during your last pregnancy or within 42 days after the birth?	Yes 1<br>No 2	→ ask Q91
81	On average, how many time have you received phone calls?	No. of phone call [ ][ ]<br>Don't remember 98	
82	What language was used by the call centre agent?	Urdu 1<br>English 2<br>Punjabi 3<br>Sindhi 4	

83	Did the MSS call at a time that was convenient for you to discuss?	Yes 1<br>No 2	→ ask Q85
84	If no, what should be the best time to call you for the information/counseling?	Time _ _ : _ _	
85	Were you comfortable with the following actions made by MSS?<br><br>Timing of phone call<br>Length of phone call<br>Language of call centre agent<br>Number of phone calls	**Yes** **No**<br>1 2<br>1 2<br>1 2<br>1 2	
86	What kind of information was provided by MSS call centre agent?<br><br>**[MULTIPLE RESPONSES ALLOWED]**	Minimum no. of antenatal care visits 1<br>Timing of antenatal care visits 2<br>How many tetanus toxoid injections required during pregnancy 3<br>Provide information about nutrition supplementary during pregnancy 4<br>Highlighted danger signs of pregnancy 5<br>What to do if any danger signs of pregnancy appeared 6<br>Where to go in case of emergency during pregnancy 7<br>How and what to plan for delivery 8<br>Arrangement of necessary things (money, blood donor, travel, pre-identify skilled provider and health facility) for delivery 9<br>Provided information about breastfeeding (When should start, till what age) 10<br>Counseled on child immunization 11<br>Highlighted newborn danger signs 12<br>Number of immunization should a child receive 13<br>Highlighted essential newborn care practices 14<br>Highlighted importance of post-partum family planning/birth spacing 15<br>Place where to obtain FP method after delivery 16<br>Minimum no. of postnatal care visits 17<br>Timing of postnatal care visits 18<br>Manage/identify postnatal complications 19<br>Counseled on danger signs after delivery 20<br>**Others (specify)**<br>l) ________________________<br>m) ________________________<br>n) ________________________<br>o) ________________________	
87	Are you satisfied with the information/counselling provided by the MSS via phone calls?	Yes – Completely satisfied 1<br>Yes – Somewhat satisfied 2<br>Neutral/Just fine 3<br>No – Somewhat unsatisfied 4<br>No – completely unsatisfied 5	

88	Are you satisfied with the behavior of MSS call centre agent?	Yes – Completely satisfied 1<br>Yes – Somewhat satisfied 2<br>Neutral/Just fine 3<br>No – Somewhat unsatisfied 4<br>No – completely unsatisfied 5	
89	Was the call centre agent able to understand your problems/concerns?<br><br>Probe: Did you discuss anything with the agent?	Yes 1<br>No 2	
90	How useful/effective was the information/counselling provided by MSS via phone calls?	Completely useful/effective 1<br>Somewhat useful/effective 2<br>Neutral/Just fine 3<br>Somewhat useless/ineffective 4<br>Completely useless/ineffective 5	
Now, I would like to ask some questions about overall experience regarding Voice Messages/SMS/Phone Calls			
91	Were things easy to learn?	Yes 1<br>No 2	
92	**[ask if participant received SMS/Voice Messages, otherwise ask Q203]**<br><br>Some women received an interactive SMS/voice messages from the senders. These interactive SMS/voice messages enable women to get the specific or desire information using reply function at the time of receiving SMS/voice messages or directly instantly talk to someone for information.<br><br>So, would you prefer to ask questions using reply function/option to interact with the senders?	Yes 1<br>No 2	
93	If you ask to select other method/option of receiving information, what would you prefer?<br><br>**[MULTIPLE ANSWER ALLOWED]**	SMS 1<br>Voice Messages 2<br>Phone calls 3<br>None 4	
94	Is there any recommendations you would want to change?	Yes 1<br>No 2	→ ask Q96
95	If yes, what would you recommend?	____________________________________<br>____________________________________<br>____________________________________<br>_________________________________	
96	Did anything bad happen to you as a result of being in this study?<br>e.g. family conflict, violence etc.	Yes 1<br>No 2	→ ask 100

97	If so, what happened?	Threats of abuse including use of a weapon 1<br>Slapping, pushing, no injuries and/or lasting pain 2<br>Punching, kicking, bruises, cuts and/or continuing pain 3<br>Beating up, severe contusions, burns, broken bones 4<br>Head injury, internal injury, permanent injury 5<br>Use of weapon, wound from weapon 6	
98	If yes, by whom?	Husband 1<br>In laws 2<br>Other	
99	How many times?		
100	Would you like to have further information or counselling?	Yes 1<br>No 2	→ ask 102
101	What would you like to know further?	____<br>____<br>____<br>____	
102	Would you recommend this intervention to a friend?	Yes 1<br>No 2	→ ask 104
103	If not, why not?	____<br>____<br>____<br>____	

SECTION 5: SOCIAL SUPPORT

Now I would like to ask some questions to know about the people give personal support to you. By supportive person we mean a person a) - who is helpful, b) - who will listen to you, or c)-who will back you up when you are in trouble. We will read out from a list one by one and you have to decide how much each person (or group of persons) is supportive for you AT THIS TIME IN YOUR LIFE. Interviewer: circle the codes of the responses.

104	How supportive is your husband now-a-days?	None 0<br>Some 1<br>A lot 2	
105	Do you have elder children/child above 18 years of age?	Yes 1<br>No 2	→ ask 107
106	How supportive are your elder children/child now-a-days?	None 0<br>Some 1<br>A lot 2	
107	Are your parents (mother/father) alive?	Yes 1<br>No 2	→ ask 109
108	How supportive are your parent now-a-days?	None 0<br>Some 1<br>A lot 2	
109	Do you have siblings?	Yes 1<br>No 2	→ ask 111
110	How supportive are your siblings now-a-days?	None 0<br>Some 1<br>A lot 2	
111	Do you have other maternal and paternal relatives (Uncles, Aunts and cousins)?	Yes 1<br>No 2	→ ask 113

112	How supportive are your other maternal and paternal relatives (Uncles, Aunts and cousins) now-a-days?	None 0<br>Some 1<br>A lot 2	
113	Do you have relatives by marriage (e.g., mother-in- law, father-in-law, sister-in-law, and brother-in-law)[1]?	Yes 1<br>No 2	→ ask 115
114	How supportive are they now-a-days?	None 0<br>Some 1<br>A lot 2	
115	Do you have neighbours (other than relatives)?	Yes 1<br>No 2	→ ask 117
116	How supportive are your neighbours now-a-days?	None 0<br>Some 1<br>A lot 2	
117	Are you visited by workers of any government or non-government organization[2] ?	Yes 1<br>No 2	→ ask 122
118	How supportive are these workers to you now-a-days?	None 0<br>Some 1<br>A lot 2	
119	a) ______________________________	None 0<br>Some 1<br>A lot 2	
120	b) ______________________________	None 0<br>Some 1<br>A lot 2	
121	c) ______________________________	None 0<br>Some 1<br>A lot 2	
122	Are you member of any local welfare committee / religious gatherings?	Yes 1<br>No 2	→ ask 124
123	How supportive are these groups to you now-a-days?	None 0<br>Some 1<br>A lot 2	
124	Do you have other friends?	Yes 1<br>No 2	→ ask 126
125	How supportive are your other friends now-a-days?	None 0<br>Some 1<br>A lot 2	
126	Do you have at least one particular person whom you trust and to whom you can go with personal difficulties?	Yes 1<br>No 2	→ ask 128
127	IF yes, which of the above types of person are they? (write down from the ST1a-10a category)	______________________________________	

[1] Including parents in law, brother and sisters -in-laws
[2] Includes any frontline health or social worker

**SECTION 6: DOMESTIC VIOLENCE ASSESSMENT**			
**Read to respondent**: *"Now I would like to ask you questions about some other important aspects of a woman's life. I know that some of these questions are very personal. Let me assure you that your answers are completely confidential and will not be told to anyone, and no one else will know that you were asked these questions. You do not have to answer these questions if you do not want to; you can just tell me to skip to the next question.*<br>*Read questions to respondent exactly as written. Do not read out the response options. Circle the number corresponding closest to the respondent's answer. All questions must be answered.*			
128	Have you ever been emotionally or physically abused by your husband or someone close to you?	No 1<br>Yes 2	→ ask 132
129	Within the six months, have you been hit, slapped, kicked or otherwise physically harmed by someone?	No 1<br>Yes 2	→ ask 132
130	If yes, by whom?	Husband 1<br>In laws 2<br>Other 3	
131	How many times?		
132	Since you've been pregnant, were you hit, slapped, kicked, or otherwise physically harmed by someone?	No 1<br>Yes 2	→ ask 138
133	If yes, by whom?	Husband 1<br>In laws 2<br>Other 3	
134	How many times?		
135	Which part you were hit most on your body?<br><br>[Only one answer is allowed]	Head 1<br>Face 2<br>Chest/Breast 3<br>Stomach/Abdomen 4<br>Upper back 5<br>Buttock 6<br>Leg 7	
136	What instrument used for violence?	Using hand 1<br>Using instrument like knife/wood/pipe etc 2<br>Others (specify)___________________________	
137	What was the severity of violence?	No / Yes<br>Injuries and/or lasting pain 1 2<br>Bruises, cuts and/or continuing pain 1 2<br>Severe contusions, burns, broken bones 1 2<br>Internal injury, permanent injury 1 2	
138	In the last six months, has your husband physically forced you to have sexual intercourse with him even when you did not want to?	No 1<br>Yes 2	→ ask 140
139	How many times?		
140	Are you afraid of your husband?	No 1<br>Yes 2	
	**THANK YOU FOR YOUR PARTICIPATION**		
