# Supplementary material for: A study protocol for an mHealth, multi-centre randomized control trial to promote use of postpartum contraception amongst rural women in Punjab, Pakistan
Source: BMC Pregnancy Childbirth. 2019 Aug 8;19:283. doi: 10.1186/s12884-019-2427-z (PMC6686472; doi:10.1186/s12884-019-2427-z)
Supplement: Supplementary file 3 — Baseline Questionnaire for the Study (Urdu). (PDF 7650 kb) [file 12884_2019_2427_MOESM3_ESM.pdf]

شرکت کنندہ کا انفرادی کوڈ شیٹ سے دیکھ کر لکھیں:					

Using mhealth for promoting use of postpartum contraception (PPIUCD study)

نمبر	سوالات	جوابات	ترتیب
سیکشن ایک: سماجی و معاشی حیثیت			
	موجودہ وقت نوٹ کریں	گھنٹے ____ منٹ ____	
سب سے پہلے میں آپ کے اور آپ کے شوہر کے بارے میں چند سوالات کرونگی۔			
نمبر	سوالات	جوابات	ترتیب
101	آپ کی عمر کتنی ہے (سالوں میں)؟	سال ____	
102	آپ کے شوہر کی عمر کتنی ہے (سالوں میں)؟	سال ____	
103	شادی کے وقت آپ کی عمر کتنی تھی؟	سال ____	
104	آپ نے زیادہ سے زیادہ کہاں تک تعلیم حاصل کی ہے؟	[1] غیر تعلیم یافتہ [لکھ پڑ نہیں سکتی]<br>[2] سادہ جملہ لکھ اور پڑھ سکتی ہیں اور نمبر یا رقم کو جمع کر سکتی ہیں<br>[3] پرائمری [1 سے 5 جماعت]<br>[4] مڈل [6 سے 8 جماعت]<br>[5] سیکنڈری [8 سے 10 جماعت]<br>[6] انٹر [11 سے 12 جماعت]<br>[7] گریجویشن یا زیادہ [13 سے زیادہ جماعتیں]	
105	آپ کے شوہر نے زیادہ سے زیادہ کہاں تک تعلیم حاصل کی ہے؟	[1] غیر تعلیم یافتہ [لکھ پڑ نہیں سکتے]<br>[2] سادہ جملہ لکھ اور پڑھ سکتے ہیں اور نمبر یا رقم کو جمع کر سکتے ہیں<br>[3] پرائمری [1 سے 5 جماعت]<br>[4] مڈل [6 سے 8 جماعت]<br>[5] سیکنڈری [8 سے 10 جماعت]<br>[6] انٹر [11 سے 12 جماعت]<br>[7] گریجویشن یا زیادہ [13 سے زیادہ جماعتیں]	
106	آپ کا پیشہ کیا ہے، یعنی آپ گھریلو آمدنی کے لیے آپ کیا کام کرتی ہیں؟	[1] گھریلو خاتون [2] زراعت / کھیتی باڑی<br>[3] دکان داری [4] درزی کا کام<br>[5] رٹائرڈ [6] طالبِ علم<br>دیگر وضاحت کریں ____________	
107	آپ کے شوہر کا پیشہ کیا ہے، یعنی آپ کے شوہر گھریلو آمدنی کے لیے کیا کام کرتے ہیں؟	[1] بے روزگار [2] زراعت / کھیتی باڑی<br>[3] دکان داری [4] درزی کا کام<br>[5] رٹائرڈ [6] طالبِ علم<br>دیگر وضاحت کریں ____________	

108	کیا آپ لوگ (شوہر اور بچے) اس گھرانے میں اپنے سسرال والوں کے ساتھ رہتے ہیں یا علیحدہ رہتے ہیں؟	[1] مشترکہ خاندان<br>[2] انفرادی خاندان	
**سیکشن دو: تولیدی صحت**			
اب میں آپ سے پوری زندگی میں ہونے والے تمام حمل کے بارے میں پوچھنا چاہونگی۔ اس سے میری مراد یہ ہے کہ وہ تمام بچے جن کو آپ نے جنم دیا۔			
201	آپ زندگی میں کتنی مرتبہ حاملہ ہوئی ہیں؟ (موجودہ حمل کو شامل کر کے بتائیے)	حمل کی تعداد ☐	اگر '1' تو 205 پوچھیں
202	خواتین کو بعض اوقات ایسے حمل بھی ہوتے ہیں جن کے نتیجے میں زندہ بچہ پیدا نہیں ہوتا۔ یعنی حمل ضائع ہو سکتا ہے، گروایا جا سکتا ہے یا بچہ مردہ پیدا ہو سکتا ہے۔<br>کیا آپ کو کبھی ایسا حمل ہوا جس میں زندہ پیدائش نہ ہوئی ہو؟	[1] ہاں<br>[2] نہیں	204 پوچھیں
203	آپ کو کتنے حمل ہوئے جس کے نتیجے میں زندہ پیدائش نہ ہوئی ہو؟	تعداد ☐	
204	آپ کے کتنے بچے ہیں جو حیات ہیں؟	لڑکے ☐ لڑکیاں ☐	
205	جب آپ اِس دفعہ حاملہ ہوئی، تو کیا آپ اُس وقت حاملہ ہونا چاہتی تھیں، یا آپ بعد میں حمل چاہتی تھیں، یا آپ مزید بچے بالکل نہیں چاہتی تھیں؟	[1] اُس وقت حمل چاہتی تھی<br>[2] بعد میں حمل چاہتی تھی<br>[3] مزید بچے بالکل نہیں چاہتی تھی	
206	اِس بچے کی پیدائش کے بعد، آپ اگلے بچے کے لیے کتنا انتظار کرنا چاہیں گی؟	سال ☐☐<br>مہینے ☐☐<br>[0] مزید بچے نہیں چاہتی	
207	اِس بچے کی پیدائش کے بعد، آپ کے شوہر اگلے بچے کے لیے کتنا انتظار کرنا چاہیں گے؟	سال ☐☐<br>مہینے ☐☐<br>[0] مزید بچے نہیں چاہتے<br>[98] معلوم نہیں	
208	[سوال نمبر 201 چیک کریں۔ اگر حمل کی تعداد ایک سے زیادہ ہے تو یہ سوال پوچھیں، ورنہ سوال 210 پوچھیں]<br>آپ کے آخری حمل کا کیا نتیجہ تھا؟	[1] زندہ پیدائش [2] مردہ پیدائش<br>[3] حمل کا ضائع ہونا [4] اسقاطِ حمل	
209	[سوال نمبر 201 چیک کریں۔ اگر حمل کی تعداد ایک سے زیادہ ہے تو یہ سوال پوچھیں، ورنہ سوال 210 پوچھیں]<br>آپ کا آخری حمل کتنے عرصہ پہلے اختتام ہوا تھا؟	دن ☐☐<br>مہینہ ☐☐<br>سال ☐☐	
210	[سوال نمبر 204 چیک کریں۔ اگر خاتون کا کم سے کم ایک زندہ بچہ ہو تو یہ سوال پوچھیں، ورنہ اگلے سیکشن 3 پر جائیں]<br>آپ کے سب سے چھوٹے بچے کی عمر کتنی ہے؟	سال ☐☐<br>مہینے ☐☐	

211	سوال 210 میں جس بچے کی عمر بتائی ہے اس کا نام پوچھیں اور یہاں درج کریں: **نام**________________<br>[بچے کا نام لیں] سے جو بڑا بچہ ہے اس کی عمر کیا ہے؟	سال [ ][ ]<br>مہینے [ ][ ]<br>[1] ایک ہی بچہ/بچی ہے	

سیکشن تین: صحت کے حوالے سے آگاہی

اب میں آپ سے چند سوالوں کے ذریعے یہ جاننا چاہوں گی کہ حمل، بچے کی پیدائش اور پیدا ہونے والے بچے کی صحت کے حوالے سے کن باتوں کا خیال رکھنا چاہیے۔

301	کیا آپ ہمیں بتائینگی، بچے کی پیدائش کے لیے کیا ضروری انتظامات پہلے سے کرنے چاہیے؟<br>کریدیں: اور کچھ؟ **[ایک سے زیادہ جوابات ممکن ہیں]**	[1] زچگی کے لئے، صحت کے مرکز کے بارے میں پہلے سے معلومات<br>[2] زچگی کے لئے، تربیت یافتہ صحت کے فرد کے بارے میں پہلے سے معلومات<br>[3] حاملہ عورت کو صحت کے مرکز پر لے جانے کے لئے گاڑی کا پہلے سے انتظام<br>[4] گاڑی کے انتظام اور زچگی کے لئے ہونے والے اخراجات کے لئے رقم کا انتظام<br>[5] زچگی یا اس سلسلے میں ہونے والے درد کے دوران ممکنہ طور پر پیدا ہونے والی ہنگامی صورت کے لیے خون کا پہلے سے انتظام<br>[6] حمل کے دوران تربیت یافتہ صحت کے فرد سے معائنہ کروانا<br>[98] معلوم نہیں<br>**دیگر وضاحت کریں:**<br>**الف:**____________________<br>**ب:**____________________<br>**ج:**____________________	
302	حمل کے دوران خطرے کی کون سی علامات ہو سکتی ہیں جن کے سلسلے میں صحت کے فرد (مثلاً ڈاکٹر) کی فوری ضرورت پیش آسکتی ہے؟<br>کریدیں: اور کچھ؟ **[ایک سے زیادہ جوابات ممکن ہیں]**	[1] سانس لینے میں مشکل پیش آنا<br>[2] شرم گاہ سے خون آنا<br>[3] بل پڑنا، جھٹکے لگنا یا دورے پڑنا یا بے ہوش ہو جانا / سر میں شدید درد ہونا / دُھندلا نظر آنا<br>[4] پیٹ میں شدید درد ہونا<br>[5] شرم گاہ سے غیر معمولی بدبُودار پانی کا آنا<br>[6] پیٹ میں بچے کی حرکت میں کمی آنا یا حرکت کا بالکل نہ ہونا<br>[98] معلوم نہیں<br>**دیگر وضاحت کریں:**<br>**الف:**____________________<br>**ب:**____________________<br>**ج:**____________________	

303	آپ کے خیال میں دوران حمل کم از کم کتنی بار معائنہ کروانا چاہیے؟	تعداد [ ][ ]<br>[98] معلوم نہیں	
304	حمل کا پہلا معائنہ کب کروانا چاہیے؟	[1]پہلی سہ ماہی (1 سے 3 ماہ کا حمل)<br>[2]دوسری سہ ماہی (4 سے 6 ماہ کا حمل)<br>[3]تیسری سہ ماہی (7 سے حمل کا آخری مہینہ)<br>[98] معلوم نہیں	
305	حمل کے دوران تشنج سے بچاؤ کے لیے کتنے حفاظتی ٹیکہ ٹیکے لگوانے چاہیے؟	تعداد [ ][ ]<br>[98] معلوم نہیں	
306	بچے کی پیدائش کے بعد چھ ہفتوں کے عرصے میں،ماں کے لیے خطرے کی کون سی علامات ہو سکتی ہیں جن کے سلسلے میں ڈاکٹر وغیرہ کی فوری ضرورت پیش آسکتی ہے؟<br><br>کریدیں: اور کچھ؟ **[ایک سے زیادہ جوابات ممکن ہیں]**	[1] شرم گاہ سے خوُن آنا<br>[2] سانس لینے میں مشکل پیش آنا<br>[3] بخار ہونا<br>[4] پیٹ میں درد ہونا<br>[5] سر میں شدید درد ہونا / دُھندلا نظر آنا<br>[6]بل پڑنا، جھٹکے لگنا یا دورے پڑنا یا بے ہوش ہو جانا<br>[7] شرم گاہ سے غیر معمولی بدبُودار پانی کا آنا<br>[8] ایسی کیفیت یا خیالات آنا جن سے یہ ظاہر ہو کہ ماں خود کو یا اپنے بچّے کو نقصان پہنچائے گی<br>[98] معلوم نہیں<br>**دیگر وضاحت کریں:**<br>**الف:**______________________________<br>**ب:**______________________________<br>**ج:**______________________________	
307	آپ کے خیال میں بچے کی پیدائش کے بعد کتنی مرتبہ ماں اور بچے کا معائنہ کروانا چاہیے؟	تعداد [ ][ ]<br>[98] معلوم نہیں	
308	پیدائش کے بعد چھ ہفتوں کے عرصے میں، بچے کے لیے خطرے کی کون سی علامات ہو سکتی ہیں جن کے سلسلے میں، ڈاکٹر وغیرہ کی فوری ضرورت پیش آسکتی ہے؟<br><br>کریدیں: اور کچھ؟ **[ایک سے زیادہ جوابات ممکن ہیں]**	[1] سانس لینے میں مشکل پیش آنا<br>[2]بل پڑنا، جھٹکے لگنا یا دورے پڑنا یا بے ہوش ہو جانا<br>[3]جِلد اور منہ کی جھلیوں کا رنگ نیلا ہو جانا<br>[4]رنگت کا زرد (پیلا) ہو جانا<br>[5]سُستی / کاہلی<br>[6]جِلد اور منہ کی جھلیوں کا رنگ نیلا ہو جانا<br>[7]چھوُنے پر جسم گرم محسوس ہونا (بخار)<br>[8]خوُن کا اخراج ہونا	

		[9]شدید یرقان (پیلیا / جِلد اور آنکھوں کا رنگ پیلا ہو جاتا ہے)<br>[10]بچّہ دُودھ نہیں پیتا یا بہت کم پیتا ہے<br>[11]دست ہونا<br>[12]مستقل طور پر اُلٹیاں ہونا یا پیٹ کا پُھول جانا<br>[13]ناف میں پَس پڑ جانا یا، آنکھوں یا جِلد کا سُرخ ہو جانا<br>[14]ہاتھوں، ٹانگوں یا جوڑوں میں سُوجن ہونا<br>[98] معلوم نہیں<br>**دیگر وضاحت کریں:**<br>**الف:**__________________<br>**ب:**__________________<br>**ج:**__________________	
309	پیدائش کے فوراً بعد بچے کو گرم رکھنے کے لیے کیا کرنا چاہیے؟	[1] بچے کو گرم کپڑے میں لپیٹنا چاہیے<br>[2] بچے کو ماں کی برہنہ چھاتی کے ساتھ لگانا چاہیے<br>[98] معلوم نہیں<br>**دیگر وضاحت کریں:**________________	
310	پیدائش کے بعد بچے کو ماں کا دودھ کب سے پلانا چاہیے؟	منٹ ☐☐<br>گھنٹے ☐☐<br>دن ☐☐<br>[0] فوراً<br>[98] معلوم نہیں	
311	کس عمر تک بچے کو صرف اور صرف ماں کا دودھ پلانا چاہیے؟	بچے کی عمر مہینوں میں ☐☐<br>[98] معلوم نہیں	
312	یہ بتائیے، پیدائش کے بعد بچے کو بیماریوں سے بچاو کے لیے پہلا حفاظتی ٹیکہ کب لگانا چاہیے؟	منٹ ☐☐<br>گھنٹے ☐☐<br>دن ☐☐<br>[0] پیدائش کے فوری بعد<br>[98] معلوم نہیں	

سیکشن چار: حمل، بچے کی پیدائش اور اس کے بعد کی دیکھ بھال

اب میں آپ سے دیکھ بھال کے بارے میں جاننا چاہوں گی جو آپ نے (نام) کے وقت حمل کے دوران، (نام) کی پیدائش اور اسکے بعد 42 دِنوں کے عرصے میں حاصل کی۔

401	کیا آپ گزشتہ حمل کے دوران حمل کی دیکھ بھال یا معائنے کے لیے کسی کے پاس گئی تھیں؟	[1]ہاں<br>[2] نہیں	← 405 پوچھیں
402	اُس حمل کے دوران آپ نے کتنی بار اپنا چیک اپ / معائنہ کروایا؟	تعداد ☐☐<br>[98] معلوم نہیں	

403	گزشتہ حمل کے دوران چیک اپ یا معائنہ کہاں سے کروایا تھا؟	[1]اپنے گھر میں [2]کسی اور کے گھر میں<br>[3]سرکاری ہسپتال [4]دیہی مرکز صحت<br>[5]بنیادی مرکز صحت [6]ڈسپنسری<br>[7]زچہ بچہ سنٹر [8] فلاحی مرکز / فیملی ویلفیئر سنٹر<br>[9]نجی ہسپتال یا کلینک [10]ہومیوپیتھ کا کلینک<br>[11]سورج سنٹر<br>دیگر وضاحت کریں ____________________	
404	گزشتہ حمل کے دوران چیک اپ / معائنہ کے لیے آپ کس کے پاس گئیں تھیں یعنی آپ کا معائنہ کس نے کیا تھا؟	[1]ڈاکٹر [2]نرس [3]میڈوائف<br>[4]لیڈی ہیلتھ وزیٹر [5]لیڈی ہیلتھ ورکر<br>[6]حکیم / ہومیوپیتھک ڈاکٹر [7]ڈسپینسر / کمپوڈر<br>[8]غیر تربیت یافتہ دائی<br>دیگر وضاحت کریں ____________________	
405	**نوٹ 1:** خاتون سے سب سے چھوٹے بچے کا نام پوچھیں یا آخری پیدا ہونے والے بچے کا نام پوچھیں اور نام لیکر سوال کریں۔<br>[بچے کا نام] کی پیدائش کہاں ہوئی تھی؟<br>**نوٹ 2:** اگر خاتون کی گزشتہ زچگی میں بچے کی فوتگی ہو گئی تھی یا بچہ بعد میں فوت ہو گیا تھا تو یہ سوال کریں۔<br>فوت ہونے والے بچے کی پیدائش کہاں ہوئی تھی؟	[1]اپنے گھر میں [2]کسی اور کے گھر میں<br>[3]سرکاری ہسپتال [4]دیہی مرکز صحت<br>[5]بنیادی مرکز صحت [6] زچہ بچہ سنٹر<br>[7] نجی ہسپتال یا کلینک [8]ہومیوپیتھ کا کلینک<br>[9]سورج سنٹر<br>دیگر وضاحت کریں ____________________	
406	[بچے کا نام] کی پیدائش بڑے آپریشن کے ذریعے ہوئی تھی جس میں پیٹ کاٹ کر بچہ باہر نکال لیتے ہیں؟	[1]ہاں<br>[2]نہیں	
407	[بچے کا نام] کی پیدائش کروانے میں کس نے مدد کی تھی؟	[1]خود سے زچگی ہوئی [2]ڈاکٹر [3]نرس<br>[4] میڈوائف [5]لیڈی ہیلتھ وزیٹر<br>[6]غیر تربیت یافتہ دائی [7]فیملی ویلفیئر ورکر [8] حکیم<br>دیگر وضاحت کریں ____________________	
408	[بچے کا نام] کی پیدائش کے بعد، کیا آپ یا آپکے شوہر نے حمل سے بچاؤ یا اس میں تاخیر کے لیے کوئی طریقہ استعمال کیا تھا؟	[1]ہاں<br>[2]نہیں ────────────	◄501 پوچھیں
409	کون سا طریقہ آپ یا آپ کے شوہر نے استعمال کیا تھا؟	[1]چھلہ [2]امپلانٹ<br>[3]مانع حمل کی گولیاں [4]کنڈوم<br>[5]مانع حمل ٹیکہ / انجکشن [6]وقتی پرہیز<br>[7]عزل<br>دیگر وضاحت کریں ______________	

410	آپ نے یہ طریقہ گزشتہ زچگی/بچے کی پیدائش کے کتنے عرصے بعد حاصل کیا تھا؟	کتنے دن بعد<br>کتنے مہینے بعد	☐☐<br>☐☐	
411	آپ نے یہ طریقہ کہاں سے حاصل کیا؟	[1]سرکاری ہسپتال یا کلینک<br>[3]بنیادی مرکز صحت<br>[5] نجی ہسپتال یا کلینک<br>[7] ہومیو پیتھ کا کلینک<br>[9] شوہر کو معلوم ہے<br>دیگر وضاحت کریں________	[2]دیہی مرکز صحت<br>[4]زچہ بچہ سنٹر<br>[6]سورج سنٹر<br>[8] دوا کی دکان/فارمیسی	

سیکشن پانچ: خاندانی منصوبہ بندی کے بارے میں معلومات

اب میں آپ سے خاندانی منصوبہ بندی کے حوالے سے آپ کی آگاہی اور ماضی میں اس کے استعمال کے بارے میں چند سوالات پوچھنا چاہو نگی۔

	فیملی پلاننگ کا طریقہ	کیا آپ نے کبھی [طریقہ کا نام] کے بارے میں سنا ہے؟		فیملی پلاننگ کے طریقہ کے بارے میں علم	
501	مانع حمل گولیاں	[1]ہاں	[2]نہیں ↓	اگر آپ ایک گولی کھانا بھول جائیں اور اگلے دن یاد آجائے تو آپ کیا کرینگی؟	[1]دو گولیاں کھالو نگی<br>[2]دیگر<br>[9]معلوم نہیں
502	چھلہ/آئی۔یو۔سی۔ڈی	[1]ہاں	[2]نہیں ↓	کیا آپ مجھے بتاینگی کہ چھلہ/آئی۔یو۔سی۔ڈی کہاں رکھا جاتا ہے؟	[1] بچہ دانی میں<br>[2]دیگر<br>[9]معلوم نہیں
503	انجکشن/ٹیکہ	[1]ہاں	[2]نہیں ↓	کیا آپ مجھے بتاینگی کہ انجکشن/ٹیکہ کہاں لگایا جاتا ہے؟	[1]بازوں میں<br>[2]دیگر<br>[9]معلوم نہیں
504	امپلانٹ	[1]ہاں	[2]نہیں ↓	کیا آپ مجھے بتاینگی کہ امپلانٹ کہاں رکھا جاتا ہے؟	[1]بازوں میں<br>[2]دیگر<br>[9]معلوم نہیں
505	کنڈوم	[1]ہاں	[2]نہیں ↓	آپ ایک کنڈوم کتنی مرتبہ استعمال کر سکتی ہیں؟	[1]ایک مرتبہ<br>[2]دیگر<br>[9]معلوم نہیں
506	عورت کی نل بندی	[1]ہاں	[2]نہیں ↓	ایک مرتبہ نل بندی کروانے کے بعد کیا آپ دوبارہ کبھی حاملہ ہوسکتی ہیں؟	[0]نہیں<br>[1]دیگر<br>[9]معلوم نہیں
507	مردوں کی نس بندی	[1]ہاں	[2]نہیں ↓		
508	وقتی پرہیز کا طریقہ	[1]ہاں	[2]نہیں ↓		
509	عزل اخراج	[1]ہاں	[2]نہیں ↓		
510	ماں کے دودھ کے ذریعے	[1]ہاں	[2]نہیں ↓		
511	ہنگامی مانع حمل کے طریقے	[1]ہاں	[2]نہیں ↓		
512	دیگر وضاحت________	[1]ہاں	[2]نہیں ↓		

513	کیا آپ نے کبھی کوئی چیز استعمال کی یا کوئی طریقہ آزمایا ہے تاکہ حمل میں تاخیر یا اس سے بچا جا سکے؟	[1]ہاں ←<br>[2]نہیں [3]ابھی فیصلہ نہیں کیا	515 پوچھیں
514	ماضی میں کبھی کوئی بھی طریقے استعمال نہ کرنے کی سب سے اہم وجہ کیا ہے؟	[1]مزید بچوں کی خواہش<br>[2]کم ملاپ / بے قاعدہ ملاپ<br>[3]ماہواری کا بند ہونا / آپریشن سے بچہ دانی نکال دینا<br>[4]بانجھ پن<br>[5] بچے کی پیدائش کے بعد ماہواری بند ہو گئی<br>[6]دودھ پلانا<br>[7]جو اللہ کی رضا<br>[8]فیملی پلاننگ کی مخالف<br>[9]شوہر کی مخالفت<br>[10]دوسرے لوگوں کی مخالفت<br>[11]مذہبی وجوہات<br>[12]کسی طریقے سے واقف نہیں<br>[13] طریقہ حاصل کرنے کا ذریعے معلوم نہیں<br>[14]صحت کے خدشات<br>[15]مضر اثرات کا خوف<br>[16]بہت دور ہے<br>[17]بہت مہنگا<br>[18]استعمال میں مشکل<br>دیگر وضاحت کریں________	
اب میں آپ سے مستقبل میں مانع حمل کے استعمال کے بارے میں چند سوالات پوچھنا چاہوں گی۔			
515	اِس بچے کی پیدائش کے بعد، کیا آپ اگلے حمل میں تاخیر یا بچنے کے لیے خاندانی منصوبہ بندی کا کوئی طریقہ استعمال کرنے کا ارادہ رکھتی ہیں؟	[1]ہاں ←<br>[2]نہیں	517 پوچھیں
516	مستقبل میں طریقے استعمال نہ کرنے کی سب سے اہم وجہ کیا ہے؟	[1]کم ملاپ / بے قاعدہ ملاپ<br>[2]دودھ پلانا<br>[3]جو اللہ کی رضا<br>[4]مزید بچوں کی خواہش<br>[5]فیملی پلاننگ کی مخالف<br>[6]شوہر کی مخالفت<br>[7]دوسرے لوگوں کی مخالفت<br>[8]مذہبی وجوہات	

		[9] کسی طریقے سے واقف نہیں<br>[10] طریقہ حاصل کرنے کا ذریعے معلوم نہیں<br>[11] صحت کے خدشات<br>[12] مضر اثرات کا خوف<br>[13] بہت دور ہے<br>[14] بہت مہنگا<br>[15] استعمال میں مشکل<br>[98] معلوم نہیں / بتا نہیں سکتی	
اب میں آپ سے مانع حمل کے بارے میں تصورات جاننے کیلئے چند سوالات پوچھوں گیں			
	**ارادہ**		
517	کیا آپ بچوں میں وقفے کے لئے فیملی پلاننگ کا طریقہ اپنانے کی سوچ رکھتی ہیں؟	[1] بالکل نہیں [2] کچھ خاص نہیں<br>[3] نہ ہاں نہ نہیں<br>[4] کچھ حد تک ہاں [5] مکمل طور پر ہاں	
518	کیا آپ بچوں میں وقفے کے لیے ڈاکٹری طریقے اپنانا چاہتی ہیں؟	[1] بالکل نہیں [2] کچھ خاص نہیں<br>[3] نہ ہاں نہ نہیں<br>[4] کچھ حد تک ہاں [5] مکمل طور پر ہاں	
519	کیا آپ بچوں میں وقفے کے لیے ڈاکٹری طریقے اپنانے کا ارادہ رکھتی ہیں؟	[1] بالکل نہیں [2] کچھ خاص نہیں<br>[3] نہ ہاں نہ نہیں<br>[4] کچھ حد تک ہاں [5] مکمل طور پر ہاں	
	**مثبت رویہ**		
520	کیا آپ بچوں میں وقفے کے لئے ڈاکٹری طریقوں کے استعمال کو کار آمد سمجھتی ہیں؟	[1] بالکل نہیں [2] کچھ خاص نہیں<br>[3] نہ ہاں نہ نہیں<br>[4] کچھ حد تک ہاں [5] مکمل طور پر ہاں	
521	کیا آپ بچوں میں وقفے کے لئے ڈاکٹری طریقوں کے استعمال کو عقل مندانہ عمل سمجھتی ہیں؟	[1] بالکل نہیں [2] کچھ خاص نہیں<br>[3] نہ ہاں نہ نہیں<br>[4] کچھ حد تک ہاں [5] مکمل طور پر ہاں	
522	کیا آپ بچوں میں وقفے کے لئے ڈاکٹری طریقوں کے استعمال کو فائدہ مند سمجھتی ہیں؟	[1] بالکل نہیں [2] کچھ خاص نہیں<br>[3] نہ ہاں نہ نہیں<br>[4] کچھ حد تک ہاں [5] مکمل طور پر ہاں	
523	اگر قدرتی طور پر بچوں میں وقفہ نہ ہو تو ڈاکٹری طریقہ بچوں میں وقفے کے لیے موثر ہے؟	[1] بالکل نہیں [2] کچھ خاص نہیں<br>[3] نہ ہاں نہ نہیں<br>[4] کچھ حد تک ہاں [5] مکمل طور پر ہاں	

	**غلط فہمیاں اور خدشات**		
524	کیا آپ کے خیال میں بچوں میں وقفے کے لیے ڈاکٹری طریقہ استعمال کرنے سے آپ کی معمولی طبعیت خراب ہو سکتی ہے؟	[5] بالکل نہیں<br>[4] کچھ خاص نہیں<br>[3] نہ ہاں نہ نہیں<br>[2] کچھ حد تک ہاں<br>[1] مکمل طور پر ہاں	
525	کیا آپ کے خیال میں بچوں وقفے کے لیے ڈاکٹری طریقے استعمال کرنے سے آپ شدید بیمار ہو جائیں گے؟	[5] بالکل نہیں<br>[4] کچھ خاص نہیں<br>[3] نہ ہاں نہ نہیں<br>[2] کچھ حد تک ہاں<br>[1] مکمل طور پر ہاں	
526	آپ کے خیال میں کیا بچوں میں وقفے کے لیے ڈاکٹری طریقہ استعمال کرنا صحت کے لئے نقصان دے ہے؟	[5] بالکل نہیں<br>[4] کچھ خاص نہیں<br>[3] نہ ہاں نہ نہیں<br>[2] کچھ حد تک ہاں<br>[1] مکمل طور پر ہاں	
527	اگر آپ کو بچوں میں وقفے کا ڈاکٹری طریقہ استعمال کرنے سے کوئی بیماری ہو جائے تو کیا آپ کے خیال میں، آپ کو اس کے علاج پر بہت پیسے خرچ کرنے پڑیں گے؟	[5] بالکل نہیں<br>[4] کچھ خاص نہیں<br>[3] نہ ہاں نہ نہیں<br>[2] کچھ حد تک ہاں<br>[1] مکمل طور پر ہاں	
	**سماجی اقدار**		
528	کیا آپ کے قریبی لوگ بچوں میں وقفے کے لئے ڈاکٹری طریقے کے استعمال کو اچھا سمجھتے ہیں؟	[1] بالکل نہیں<br>[2] کچھ خاص نہیں<br>[3] نہ ہاں نہ نہیں<br>[4] کچھ حد تک ہاں<br>[5] مکمل طور پر ہاں	
529	کیا آپ کے خیال میں آپ کے علاقے کے لوگ بچوں میں وقفے کے لیے ڈاکٹری طریقوں کی حمایت کرتے ہیں؟	[1] بالکل نہیں<br>[2] کچھ خاص نہیں<br>[3] نہ ہاں نہ نہیں<br>[4] کچھ حد تک ہاں<br>[5] مکمل طور پر ہاں	
530	کیا آپ کے خیال میں آپ کے قریبی لوگ ان عورتوں کو سیانا سمجھتے ہیں جو بچوں میں وقفے کے لیے ڈاکٹری طریقے استعمال کرتی ہیں؟	[1] بالکل نہیں<br>[2] کچھ خاص نہیں<br>[3] نہ ہاں نہ نہیں<br>[4] کچھ حد تک ہاں<br>[5] مکمل طور پر ہاں	
531	آپ کے قریبی / جان پہچان والے لوگوں کا خیال ہے آپ کو بچوں میں وقفے کے لیے ڈاکٹری طریقہ استعمال کرنا چاہیے؟	[1] بالکل نہیں<br>[2] کچھ خاص نہیں<br>[3] نہ ہاں نہ نہیں<br>[4] کچھ حد تک ہاں<br>[5] مکمل طور پر ہاں	
	**ذاتی اختیار**		
532	کیا بچوں میں وقفے کے لئے ڈاکٹری طریقہ اپنانے کا فیصلہ آپ کے اختیار میں ہے؟	[1] بالکل نہیں<br>[2] کچھ خاص نہیں<br>[3] نہ ہاں نہ نہیں<br>[4] کچھ حد تک ہاں<br>[5] مکمل طور پر ہاں	

533	اگر آپ کو بچوں میں وقفے کے لیے ڈاکٹری طریقے استعمال کرنا ہو تو کیا اس میں آپ کو دشواری ہو گی؟	[1] بالکل نہیں<br>[2] کچھ خاص نہیں<br>[3] نہ ہاں نہ نہیں<br>[4] کچھ حد تک ہاں<br>[5] مکمل طور پر ہاں	
534	کیا آپ کے لئے، بچوں میں وقفے کے لئے ڈاکٹری طریقہ اپنانا آسان ہے؟	[1] بالکل نہیں<br>[2] کچھ خاص نہیں<br>[3] نہ ہاں نہ نہیں<br>[4] کچھ حد تک ہاں<br>[5] مکمل طور پر ہاں	

سیکشن چھ: گھرانے میں فیصلہ سازی

601: جواب دھندہ کیلئے پڑھیئے: اب میں آپ سے گھریلو فیصلہ سازی کے حوالے سے کچھ سوالات پوچھوں گی۔ میں ایک ایک کر کے روز مرہ کے مختلف معاملات کے بارے میں پوچھونگی، آپ نے بتانا ہے کہ عام طور پر ان کی فیصلہ سازی میں کون شامل ہوتا ہے: آپ خود کرتی ہیں، صرف آپ کے شوہر، صرف آپ کی ساس یا سسر، یا کوئی اور فرد۔ اگر ایک سے زیادہ افراد مل کر فیصلہ کرتے ہیں تو آپ ایک سے زیادہ لوگوں کے بارے میں بھی بتا سکتی ہیں۔

ہدایت: **[ایک سے زیادہ جوابات ممکن ہیں]**۔

تفصیل	خود (شرکت کنندہ)	شوہر	ساس	سسر	کوئی اور فرد
۱۔ چھوٹا خرچہ جیسے ٹوتھ پیسٹ، صابن، سبزی / دودھ / برتن وغیرہ کی خریداری کا فیصلہ کون کرتا ہے؟	☐	☐	☐	☐	☐
۲۔ بڑا خرچہ جیسے ٹی وی فرج وغیرہ کی خریداری کا فیصلہ کون کرتا ہے؟	☐	☐	☐	☐	☐
۳۔ عورتوں کی اشیاء مثلاً کپڑے زیورات وغیرہ کی خریداری کا فیصلہ کون کرتا ہے؟	☐	☐	☐	☐	☐
۴۔ آپ کی گھر سے باہر ملازمت کا فیصلہ کون کرتا ہے؟	☐	☐	☐	☐	☐
۵۔ جائیداد / کاشت کاری کے لئے زمین کی خرید و فروخت کا فیصلہ کون کرتا ہے؟	☐	☐	☐	☐	☐
۶۔ بچوں کے کپڑے کی خریداری کا فیصلہ کون کرتا ہے؟	☐	☐	☐	☐	☐
۷۔ اگر بچے بیمار ہوں تو کہاں لے جایا جائے یہ فیصلہ کون کرتا ہے؟	☐	☐	☐	☐	☐
۸۔ اگر آپ بیمار ہوں تو کہاں لے جایا جائے یہ فیصلہ کون کرتا ہے؟	☐	☐	☐	☐	☐
۹۔ دواوں کی خریداری کا فیصلہ کون کرتا ہے؟	☐	☐	☐	☐	☐
۱۰۔ بچوں کی تعلیم کے لیے فیصلہ کون کرتا ہے؟	☐	☐	☐	☐	☐
۱۱۔ رشتے داروں سے ملاقات (کب اور کہاں)، اس کا فیصلہ کون کرتا ہے؟	☐	☐	☐	☐	☐
۱۲۔ آپ کے بچوں کی تعداد کے بارے میں فیصلہ کون کرتا ہے؟	☐	☐	☐	☐	☐
۱۳۔ وقفے کے لیے خاندانی منصوبہ بندی کے طریقوں کے استعمال کرنے یا نہ کرنے کے حوالے سے فیصلہ کون کرتا ہے؟	☐	☐	☐	☐	☐

602	اپنی صحت کی دیکھ بھال حاصل کرنے کے لیے کیا آپ با آسانی اکیلے اپنے گھر سے باہر جا سکتی ہیں؟	[1] ہاں ←<br>[2] نہیں	701 پوچھیں

603	اگر نہیں، تو عموماً علاج کے لیے آپ کے ہمراہ کون ہوتا ہے یا آپ عموماً کس کے ساتھ علاج کے لیے باہر جاتی ہیں؟	[1]اکیلے [2]شوہر کے ہمراہ<br>[3]ساس کے ہمراہ<br>دیگر وضاحت کریں___________	
**سیکشن سات: سماجی و آبادیاتی معلومات**			
اب میں آپ سے چند سوالات آپ کے رہن سہن کے حوالے سے کرونگی۔ میں سمجھتی ہوں کہ ان کا تعلق براہ راست آپ کی صحت سے نہیں ہے لیکن یہ سوالات ہمیں ان کمیونٹی میں رہنے والے لوگوں کے رہن سہن کے بارے میں مدد دیگا۔ برائے مہربانی ایمانداری کے ساتھ سوالوں کے جوابات دیجئے تاکہ ہم اس کمیونٹی کی اچھے طریقے سے خدمت کر سکیں۔ سوالات کو ایسے ہی پڑھ کر سنائیں جیسے درج ہیں۔ جوابات نہ پڑھیں۔ شرکت کنندہ کا جواب سن کر متعلقہ جواب پر دائرہ لگائیں۔ تمام سوالات پوچھے جائیں گے۔			
701	آپ کا گھرانہ کس صوبے میں رہتا ہے؟	[1]بلوچستان [2]خیبر پختون خواہ<br>[3]پنجاب [4]سندھ	
702	گھرانے کے کتنے افراد 13 سال یا اس سے کم عمر کے ہیں؟	[1]پانچ یا پانچ سے زیادہ [2]چار<br>[3]تین [4]دو<br>[5]ایک [6]کوئی نہیں	← 704 پوچھیں
703	5 سے 13 سال کی عمر کے کتنے بچے اسکول جاتے ہیں؟	[1] کوئی بھی بچہ نہیں جاتا یا کچھ بچے اسکول جاتے ہیں<br>[2] سب بچے جاتے ہیں	
704	گھرانہ کے کتنے افراد بنیادی پیشے میں منسلک ہیں؟ مثلاً (اعلی افسران، منیجر، کسی پیشہ میں ماہر، تکنیکی یا اعلی پیشہ سے منسلک، کلرک، چیزیں فروخت کرنے والے، خدمات دینے والے، زراعت، ماہی گیری اور دوکاندار نہ ہوں)	[1]دو یا دو سے زیادہ<br>[2]ایک<br>[3]کوئی بھی نہیں	
705	گھرانہ کی سربراہ خاتون کی زیادہ سے زیادہ تعلیم کتنی ہے؟	[1]پہلی جماعت سے کم / معلوم نہیں<br>[2]گھر کی سربراہ خاتون نہیں ہے<br>[3]پہلی جماعت یا پہلی جماعت سے زیادہ	
706	گھر میں پینے کے پانی کا بنیادی ذریعہ کیا ہے؟	[1] دیگر ذرائع<br>[2] نلکا، ڈھکا ہو کنواں، موٹر لگی ہوئی ہے، پمپ، ٹیوب ویل، پائپ کے ذریعہ والا پانی	
707	گھرانہ میں کس قسم کا بیت الخلاء / ٹوائلٹ / باتھ روم استعمال کیا جاتا ہے؟	[1]کوئی بھی نہیں یا کوئی سا بھی<br>[2]فلش جس کا تعلق گھر کے نالے سے، گڑھے سے یا گٹر سے<br>[3]فلش جس کا تعلق عوامی بڑے گڑھے سے ہے	
708	کیا گھرانہ میں فریج یا فریزر ہے؟	[1]نہیں [2]ہاں	
709	کیا گھرانہ کا ذاتی ٹی وی ہے؟	[1]نہیں [2]ہاں	
710	کیا گھرانہ میں ذاتی موٹر سائیکل، سکوٹر، کار یا کوئی دوسری گاڑی ہے؟	[1]نہیں [2]ہاں	

سیکشن آٹھ: گھریلو تشدد

انٹرویو کرنے والے کے لیے ہدایات: معائنہ کریں کہ جس جگہ آپ بیٹھی ہیں یا آپ کے پاس کی جگہیں جہاں آپ کی آواز پہنچ سکتی ہو، وہاں کوئی اور موجود نہ ہو۔ رازداری کو برقرار رکھنے کے لیے اس بات کی یقین دہانی کریں کہ کوئی بھی فرد اتنے قریب نہ ہو کہ وہ آپکے سوال وجواب کو سن سکے۔ اگر آس پاس اس عمر کے بچے ہیں جو آپکی باتوں کو سمجھنے کی صلاحیت رکھتے ہیں، انھیں شائستگی سے جانے کا کہیں۔ صرف چھوٹے بچے جو آپ کی باتوں کو سمجھنے کے قابل نا ہوں، وہ بات چیت کے دوران آپ کے ہمراہ رہ سکتے ہیں۔ اُس وقت تک سوالات نہ پوچھیں، جب تک اس بات کی یقین دہانی نہ کرلیں کے آپ کے آس پاس کوئی نہیں ہے۔ اس معلومات کی اہمیت کو نظر انداز نہیں کیا جاسکتا، آپ کو رازداری رکھنے کے لیے ہر وہ عمل کرنا ہے جس کے بعد آپ سوال وجواب شروع کرسکیں۔ اگر آپ رازداری کو برقرار رکھنے میں ناکام رہیں گی تو بہت سی اہم معلومات حاصل نہیں کرپائینگی۔

جوابدہندہ کے لیئے پڑھیے: اب میں آپ سے خواتین کی زندگی کے کچھ اہم پہلوؤں کے بارے میں سوالات کرنا چاہتی ہوں۔ ہوسکتا ہے ان میں سے کچھ سوالات آپ کو بہت ہی ذاتی نوعیت کے لگیں۔ تاہم آپ کے جوابات پاکستان میں موجود خواتین کے حالات کو سمجھنے میں بے انتہا مددگار ثابت ہوں گے۔ میں آپ کو اس بات کی یقین دہانی کرواتی ہوں کہ آپ کے جوابات مکمل طور پر خفیہ رکھے جائیں گے اور ان کا ذکر کسی سے نہیں کیا جائے گا اور آپ کے گھرانے میں کسی کو یہ نہیں معلوم ہوگا کہ آپ سے کس قسم کے سوالات پوچھے گئے ہیں۔

801	کیا آپ کو کبھی بھی اپنے شوہر یا قریبی عزیز کی طرف سے جسمانی یا جذباتی تشدد کا نشانہ بنایا گیا؟	[1]ہاں<br>[2]نہیں	805 پوچھیں
802	گزشتہ 6 ماہ کے دوران، آپکو کسی نے مارا، تھپڑ مارا، ٹھوکر ماری یا کوئی جسمانی تشدد کیا؟	[1]ہاں<br>[2]نہیں	805 پوچھیں
803	اگر ہاں، تو کس نے؟	[1]شوہر [2]سسرال [3]کوئی اور	
804	کتنی دفعہ تشدد کیا؟	تعداد ☐☐	
805	جب سے آپ حاملہ ہوئی، آپکو کسی نے مارا، تھپڑ مارا، ٹھوکر ماری یا کوئی جسمانی تشدد کیا؟	[1]ہاں<br>[2]نہیں	811 پوچھیں
806	اگر ہاں، تو کس نے؟	[1]شوہر [2]سسرال [3]کوئی اور	
807	کتنی دفعہ تشدد کیا؟	تعداد ☐☐	
808	آپ کے جسم کے کس حصے پر سب سے زیادہ تشدد کیا گیا؟<br>**[صرف ایک جواب ممکن ہے، ایک سے زیادہ جواب آنے کی صورت میں سب سے اہم جواب نوٹ کریں]**	[1] سر پر<br>[2] چہرے پر<br>[3] چھاتی / سینہ پر<br>[4] پیٹ پر<br>[5] پیٹھ پر / کمر کے اوپری حصہ پر<br>[6] پشت پر، کمر کے نچلے / جسم کے پچھلے حصہ پر<br>[7]ٹانگ / گھٹنے پر	
809	تشدد کس طرح کیا گیا یعنی تشدد کے لئے کس قسم کا اوزار / ہتھیار کا استعمال کیا گیا؟	[1]ہاتھ / پیر کا استعمال کیا<br>[2] ہتھیار کا استعمال جیسے چھری / لکڑی / برتن / ڈنڈا / پائپ وغیرہ<br>دیگر (وضاحت کریں) ____________	

810	**تشدد کی نوعیت کیا تھی یعنی، کیا تشدد کرنے سے:**	**ہاں** 1 / **نہیں** 2	
	[1] چوٹ یا تھوڑے وقت تک درد رہا۔-------------------	1 — 2	
	[2] زخم، کٹ پڑ گیا یا مسلسل درد رہا۔-------------------	1 — 2	
	[3] گہرا زخم جس سے خون آیا یا ہڈی ٹوٹ گئی۔----------	1 — 2	
	[4] اندرونی چوٹ، مستقل چوٹ رہی۔----------------	1 — 2	
811	گزشتہ 6 ماہ کے دوران، کبھی آپ کے شوہر نے آپ کو زبردستی جسمانی تعلق قائم کرنے کے لیے زور دیا؟	[1] ہاں<br>[2] نہیں	← 813 پوچھیں
812	کتنی دفعہ تشدد کیا؟	تعداد ☐☐	
813	کیا آپ اپنے شوہر سے خوفزدہ رہتے ہو؟	[1] ہاں [2] نہیں	
	**آپ کے وقت کا شکریہ**		
