# Supplementary material for: A study protocol for an mHealth, multi-centre randomized control trial to promote use of postpartum contraception amongst rural women in Punjab, Pakistan
Source: BMC Pregnancy Childbirth. 2019 Aug 8;19:283. doi: 10.1186/s12884-019-2427-z (PMC6686472; doi:10.1186/s12884-019-2427-z)
Supplement: Supplementary file 4 — Follow-up Questionnaire for the Study (Urdu). (PDF 5350 kb) [file 12884_2019_2427_MOESM4_ESM.pdf]

سیکشن ایک: حمل، بچے کی پیدائش اور اس کے بعد کی دیکھ بھال

اب میں آپ سے دیکھ بھال کے بارے میں جاننا چاہوں گی جو آپ نے (نام) کے وقت حمل کے دوران، (نام) کی پیدائش اور اسکے بعد 42 دنوں کے عرصے میں حاصل کی۔

1	کیا آپ گزشتہ حمل کے دوران حمل کی دیکھ بھال یا معائنے کے لیے کسی کے پاس گئی تھیں؟	[1]ہاں<br>[2]نہیں	5 پوچھیں ←
2	اُس حمل کے دوران آپ نے کتنی بار اپنا چیک اپ / معائنہ کروایا؟	تعداد ☐☐<br>[98]معلوم نہیں	
3	آپ نے گزشتہ حمل کے دوران چیک اپ یا معائنہ کہاں سے کروایا؟	[1]اپنے گھر میں [2]کسی اور کے گھر میں<br>[3]سرکاری ہسپتال [4]دیہی مرکز صحت<br>[5]بنیادی مرکز صحت [6]ڈسپنسری<br>[7]زچہ بچہ سنٹر [8]فلاحی مرکز / فیملی ویلفیئر سنٹر<br>[9]نجی ہسپتال یا کلینک [10]ہومیوپیتھ کا کلینک<br>[11]سورج سنٹر<br>دیگر وضاحت کریں ________	
4	گزشتہ حمل کے دوران چیک اپ / معائنہ کے لیے آپ کس کے پاس گئیں تھیں یعنی آپ کا معائنہ کس نے کیا تھا؟	[1]ڈاکٹر [2]نرس<br>[3]میڈوائف [4]لیڈی ہیلتھ وزیٹر<br>[5]لیڈی ہیلتھ ورکر [6]حکیم / ہومیوپیتھک ڈاکٹر<br>[7]ڈسپنسر / کمپوڈر [8]غیر تربیت یافتہ دائی<br>دیگر وضاحت کریں ________	
5	**نوٹ:** خاتون سے نومولود بچے کا نام پوچھیں جو حال ہی میں پیدا ہوا ہے اور نام لیکر سوال کریں۔<br>[بچے کا نام] کی پیدائش کہاں ہوئی تھی؟	[1]اپنے گھر میں [2]کسی اور کے گھر میں<br>[3]سرکاری ہسپتال [4]دیہی مرکز صحت<br>[5]بنیادی مرکز صحت [6] زچہ بچہ سنٹر<br>[7] نجی ہسپتال یا کلینک [8]ہومیوپیتھ کا کلینک<br>[9]سورج سنٹر<br>دیگر وضاحت کریں ________	
6	کیا [بچے کا نام] کی پیدائش بڑے آپریشن کے ذریعے ہوئی تھی جس میں پیٹ کاٹ کر بچہ باہر نکال لیتے ہیں؟	[1]ہاں<br>[2]نہیں	
7	[بچے کا نام] کی پیدائش کروانے میں کس نے مدد کی تھی؟	[1]خود سے زچگی ہوئی [2]ڈاکٹر [3]نرس<br>[4] میڈوائف [5]لیڈی ہیلتھ وزیٹر<br>[6]غیر تربیت یافتہ دائی [7]فیملی ویلفیئر ورکر [8] حکیم<br>دیگر وضاحت کریں ________	

8	[بچے کا نام] کی پیدائش کے بعد، کیا آپ یا آپکے شوہر نے حمل سے بچاؤ یا اس میں تاخیر کے لیے کوئی طریقہ استعمال کیا تھا؟	[1]ہاں<br>[2]نہیں ←	← 12 پوچھیں
9	کون سا طریقہ آپ یا آپ کے شوہر نے استعمال کیا تھا؟	[1]چھلہ<br>[2]امپلانٹ<br>[3]مانع حمل کی گولیاں<br>[4]کنڈوم<br>[5]مانع حمل ٹیکہ / انجکشن<br>[6]عورت کی نل بندی<br>[7]وقتی پرہیز<br>[8]عزل<br>دیگر وضاحت کریں ____________	
10	آپ نے یہ طریقہ گزشتہ زچگی / بچے کی پیدائش کے کتنے عرصے بعد حاصل کیا تھا؟	کتنے دن بعد [ ][ ]<br>کتنے مہینے بعد [ ][ ]	
11	آپ نے یہ طریقہ کہاں سے حاصل کیا؟	[1]سرکاری ہسپتال یا کلینک<br>[2]دیہی مرکز صحت<br>[3]بنیادی مرکز صحت<br>[4]زچہ بچہ سنٹر<br>[5]نجی ہسپتال یا کلینک<br>[6]سورج سنٹر<br>[7] ہومیو پیتھ کا کلینک<br>[8]دوا کی دکان / فارمیسی<br>[9]شوہر کو معلوم ہے<br>دیگر وضاحت کریں ____________	
12	[بچے کا نام] کی پیدائش کے بعد، حمل سے بچاؤ یا اس میں تاخیر کے لیے کوئی بھی طریقہ استعمال نہ کرنے کی سب سے اہم وجہ کیا ہے؟	[1]مزید بچوں کی خواہش<br>[2]کم ملاپ / بے قاعدہ ملاپ<br>[3]دودھ پلانا<br>[4]جو اللہ کی رضا<br>[5]فیملی پلاننگ کی مخالف<br>[6]شوہر کی مخالفت<br>[7]دوسرے لوگوں کی مخالفت<br>[8]مذہبی وجوہات<br>[9]کسی طریقے سے واقف نہیں<br>[10]طریقہ حاصل کرنے کا ذریعے معلوم نہیں<br>[11]صحت کے خدشات<br>[12]مضر اثرات کا خوف<br>[13]بہت دور ہے<br>[14]بہت مہنگا<br>[15]استعمال میں مشکل<br>دیگر وضاحت کریں ____________	

سکشن دو: صحت کے حوالے سے آگاہی

اب میں آپ سے چند سوالوں کے ذریعے یہ جاننا چاہوں گی کہ حمل، بچے کی پیدائش اور پیدا ہونے والے بچے کی صحت کے حوالے سے کن باتوں کا خیال رکھنا چاہیے۔

13	کیا آپ ہمیں بتائینگی، بچے کی پیدائش کے لیے کیا ضروری انتظامات پہلے سے کرنے چاہیے؟<br>کریدیں: اور کچھ؟ **[ایک سے زیادہ جوابات ممکن ہیں]**	[1] زچگی کے لئے، صحت کے مرکز کے بارے میں پہلے سے معلومات<br>[2] زچگی کے لئے، تربیت یافتہ صحت کے فرد کے بارے میں پہلے سے معلومات<br>[3] حاملہ عورت کو صحت کے مرکز پر لے جانے کے لئے گاڑی کا پہلے سے انتظام<br>[4] گاڑی کے انتظام اور زچگی کے لئے ہونے والے اخراجات کے لئے رقم کا انتظام<br>[5] زچگی یا اس سلسلے میں ہونے والے درد کے دوران ممکنہ طور پر پیدا ہونے والی ہنگامی صورت کے لیے خُون کا پہلے سے انتظام<br>[6] حمل کے دوران تربیت یافتہ صحت کے فرد سے معائنہ کروانا<br>[98] معلوم نہیں<br>**دیگر وضاحت کریں:**<br>**الف:**____________________________<br>**ب:**____________________________<br>**ج:**____________________________	
14	حمل کے دوران خطرے کی کون سی علامات ہو سکتی ہیں جن کے سلسلے میں صحت کے فرد (مثلاً ڈاکٹر) کی فوری ضرورت پیش آسکتی ہے؟<br>**کریدیں: اور کچھ [ایک سے زیادہ جوابات ممکن ہیں]**	[1] سانس لینے میں مشکل پیش آنا<br>[2] شرم گاہ سے خُون آنا<br>[3] بل پڑنا، جھٹکے لگنا یا دورے پڑنا یا بے ہوش ہو جانا / سر میں شدید درد ہونا / دُھندلا نظر آنا<br>[4] پیٹ میں شدید درد ہونا<br>[5] شرم گاہ سے غیر معمولی بدبُودار پانی کا آنا<br>[6] پیٹ میں بچے کی حرکت میں کمی آنا یا حرکت کا بالکل نہ ہونا<br>[98] معلوم نہیں<br>**دیگر وضاحت کریں:**<br>**الف:**____________________________<br>**ب:**____________________________<br>**ج:**____________________________	
15	آپ کے خیال میں دوران حمل کم از کم کتنی بار معائنہ کروانا چاہیے؟	تعداد ☐☐<br>[98] معلوم نہیں	

16	دوران حمل کا پہلا معائنہ کب کروانا چاہیے؟	[1]پہلی سہ ماہی (1 سے 3 ماہ کا حمل)<br>[2]دوسری سہ ماہی (4 سے 6 ماہ کا حمل)<br>[3]تیسری سہ ماہی (7 سے حمل کا آخری مہینہ)<br>[98]معلوم نہیں	
17	دوران حمل تشنج سے بچاؤ کے لیے کتنے ٹیکے لگوانے چاہیے؟	تعداد ☐☐<br>[98]معلوم نہیں	
18	بچے کی پیدائش کے بعد چھ ہفتوں کے عرصے میں، ماں کے لیے خطرے کی کون سی علامات ہو سکتی ہیں جن کے سلسلے میں ڈاکٹر وغیرہ کی فوری ضرورت پیش آسکتی ہے؟<br><br>کریدیں: اور کچھ [ایک سے زیادہ جوابات ممکن ہیں]	[1]شرم گاہ سے خُون آنا<br>[2]سانس لینے میں مشکل پیش آنا<br>[3]بخار ہونا<br>[4]پیٹ درد ہونا<br>[5]سر میں شدید درد ہونا/ دُھندلا نظر آنا<br>[6]بل پڑنا، جھٹکے لگنا یا دورے پڑنا یا بے ہوش ہو جانا<br>[7]شرم گاہ سے غیر معمولی بد بُودار پانی کا آنا<br>[8]ایسی کیفیت یا خیالات آنا جن سے یہ ظاہر ہو کہ ماں خود کو یا اپنے بچّے کو نقصان پہنچائے گی<br>[98]معلوم نہیں<br>**دیگر وضاحت کریں:**<br>**الف:**____________________<br>**ب:**____________________<br>**ج:**____________________	
19	آپ کے خیال میں بچے کی پیدائش کے بعد کتنی مرتبہ ماں اور بچے کا معائنہ کروانا چاہیے؟	تعداد ☐☐<br>[98]معلوم نہیں	

20	پیدائش کے بعد چھ ہفتوں کے عرصے میں، بچے کے لیے خطرے کی کون سی علامات ہو سکتی ہیں جن کے سلسلے میں، ڈاکٹر وغیرہ کی فوری ضرورت پیش آسکتی ہے؟<br><br>**کریدیں: اور کچھ [ایک سے زیادہ جوابات ممکن ہیں]**	[1]سانس لینے میں مشکل پیش آنا<br>[2]بل پڑنا، جھٹکے لگنا یا دورے پڑنا یا بے ہوش ہو جانا<br>[3]جِلد اور منہ کی جھلیوں کا رنگ نیلا ہو جانا<br>[4]رنگت کا زرد (پیلا) ہو جانا<br>[5]سُستی / کاہلی<br>[6]جِلد اور منہ کی جھلیوں کا رنگ نیلا ہو جانا<br>[7]چھوُنے پر جسم گرم محسوس ہونا (بخار)<br>[8]خوُن کا اخراج ہونا<br>[9]شدید یرقان (پیلیا / جِلد اور آنکھوں کا رنگ پیِلا ہو جاتا ہے)<br>[10]بچہّ دودھ نہیں پیتا یا بہت کم پیتا ہے<br>[11]دست ہونا<br>[12]مستقل طور پر اُلٹیاں ہونا یا پیٹ کا پُھول جانا<br>[13]ناف میں پَس پڑ جانا یا، آنکھوں یا جِلد کا سُرخ ہو جانا<br>[14]ہاتھوں، ٹانگوں یا جوڑوں میں سوُجن ہونا<br>[98]معلوم نہیں<br>**دیگر وضاحت کریں:**<br>**الف:**____________________<br>**ب:**____________________<br>**ج:**____________________	
21	پیدائش کے فوراً بعد بچے کو گرم رکھنے کے لیے کیا کرنا چاہیے؟	[1]بچے کو گرم کپڑے میں لپیٹنا چاہیے<br>[2]بچے کو ماں کی برہنہ چھاتی کے ساتھ لگانا چاہیے<br>[98]معلوم نہیں<br>**دیگر وضاحت کریں:**____________________	
22	پیدائش کے بعد بچے کو ماں کا دودھ کب سے پلانا چاہیے؟	منٹ [ ][ ]<br>گھنٹے [ ][ ]<br>دن [ ][ ]<br>[0]فوراً<br>[98]معلوم نہیں	
23	کس عمر تک بچے کو صرف اور صرف ماں کا دودھ پلانا چاہیے؟	عمر مہینوں میں [ ][ ]<br>[98]معلوم نہیں	

24	یہ بتائیے، بچے کی پیدائش کے بعد احتیاطی طور پر بیماریوں سے بچاو کے لیے پہلا ٹیکا کب لگانا چاہیے؟	منٹ [ ][ ]<br>گھنٹے [ ][ ]<br>دن [ ][ ]<br>[0]پیدائش کے فوری بعد<br>[98]معلوم نہیں	

سیکشن تین: خاندانی منصوبہ بندی

اب میں آپ سے خاندانی منصوبہ بندی کے حوالے سے آپ کی آگاہی اور ماضی میں اس کے استعمال کے بارے میں چند سوالات پوچھنا چاہو نگی۔

	خاندانی منصوبہ بندی کا طریقہ	کیا آپ نے کبھی [طریقہ کا نام] کے بارے میں سنا ہے؟	کیا آپ کو کسی جگہ کا علم ہے جہاں سے خاندانی منصوبہ بندی کا طریقہ حاصل کیا جاسکتا ہے؟	
25	مانع حمل گولیاں	[1]ہاں [2]نہیں↓	[1]ہاں [2]نہیں	
26	چھلہ / آئی۔یو۔سی۔ڈی	[1]ہاں [2]نہیں↓	[1]ہاں [2]نہیں	
27	انجکشن / ٹیکہ	[1]ہاں [2]نہیں↓	[1]ہاں [2]نہیں	
28	امپلانٹ	[1]ہاں [2]نہیں↓	[1]ہاں [2]نہیں	
29	کنڈوم	[1]ہاں [2]نہیں↓	[1]ہاں [2]نہیں	
30	عورت کی نل بندی	[1]ہاں [2]نہیں↓	[1]ہاں [2]نہیں	
31	مردوں کی نس بندی	[1]ہاں [2]نہیں↓	[1]ہاں [2]نہیں	
32	وقتی پرہیز کا طریقہ	[1]ہاں [2]نہیں↓	[1]ہاں [2]نہیں	
33	عزل اخراج	[1]ہاں [2]نہیں↓	[1]ہاں [2]نہیں	
34	ماں کے دودھ کے ذریعے	[1]ہاں [2]نہیں↓	[1]ہاں [2]نہیں	
35	ہنگامی مانع حمل کے طریقے	[1]ہاں [2]نہیں↓	[1]ہاں [2]نہیں	
36	دیگر وضاحت ______	[1]ہاں [2]نہیں↓	[1]ہاں [2]نہیں	
37	کیا آپ نے کبھی کوئی چیز استعمال کی یا کوئی طریقہ آزمایا ہے تاکہ حمل میں تاخیر یا اس سے بچا جاسکے؟	[1]ہاں ←<br>[2]نہیں [3]ابھی فیصلہ نہیں کیا		← 39 پوچھیں

38	ماضی میں کبھی کوئی بھی طریقے استعمال نہ کرنے کی سب سے اہم وجہ کیا ہے؟	[1]مزید بچوں کی خواہش<br>[2]کم ملاپ / بے قاعدہ ملاپ<br>[3]ماہواری کا بند ہونا / آپریشن سے بچہ دانی نکال دینا<br>[4]بانجھ پن<br>[5]بچے کی پیدائش کے بعد ماہواری بند ہو گئی<br>[6]دودھ پلانا<br>[7]جو اللہ کی رضا<br>[8]فیملی پلاننگ کی مخالف<br>[9]شوہر کی مخالفت<br>[10]دوسرے لوگوں کی مخالفت<br>[11]مذہبی وجوہات<br>[12]کسی طریقے سے واقف نہیں<br>[13]طریقہ حاصل کرنے کا ذریعے معلوم نہیں<br>[14]صحت کے خدشات<br>[15]مضر اثرات کا خوف<br>[16]بہت دور ہے<br>[17]بہت مہنگا<br>[18]استعمال میں مشکل<br>دیگر وضاحت کریں____________________	
اب میں آپ سے مستقبل میں مانع حمل کے استعمال کے بارے میں چند سوالات پوچھنا چاہوں گی۔			
39	بچے کی پیدائش کے بعد، کیا آپ اگلے حمل میں تاخیر یا بچنے کے لیے خاندانی منصوبہ بندی کا کوئی طریقہ استعمال کرنے کا ارادہ رکھتی ہیں؟	[1]ہاں ←<br>[2]نہیں	← 41 پوچھیں

40	مستقبل میں طریقے استعمال نہ کرنے کی سب سے اہم وجہ کیا ہے؟	[1]کم ملاپ / بے قاعدہ ملاپ<br>[2]دودھ پلانا<br>[3]جو اللہ کی رضا<br>[4]مزید بچوں کی خواہش<br>[5]فیملی پلاننگ کی مخالف<br>[6]شوہر کی مخالفت<br>[7]دوسرے لوگوں کی مخالفت<br>[8]مذہبی وجوہات<br>[9]کسی طریقے سے واقف نہیں<br>[10]طریقہ حاصل کرنے کا ذریعے معلوم نہیں<br>[11]صحت کے خدشات<br>[12]مضر اثرات کا خوف<br>[13]بہت دور ہے<br>[14]بہت مہنگا<br>[15]استعمال میں مشکل<br>[98]معلوم نہیں / بتا نہیں سکتی	
اب میں آپ سے مانع حمل کے بارے میں تصورات جاننے کیلئے چند سوالات پوچھوں گیں			
	**اِرادہ**		
41	کیا آپ بچوں میں وقفے کے لئے فیملی پلاننگ کا طریقہ اپنانے کی سوچ رکھتی ہیں؟	[1]بالکل نہیں [2]کچھ خاص نہیں<br>[3]نہ ہاں نہ نہیں<br>[4]کچھ حد تک ہاں [5]مکمل طور پر ہاں	
42	کیا آپ بچوں میں وقفے کے لیے ڈاکٹری طریقے اپنانا چاہتی ہیں؟	[1]بالکل نہیں [2]کچھ خاص نہیں<br>[3]نہ ہاں نہ نہیں<br>[4]کچھ حد تک ہاں [5]مکمل طور پر ہاں	
43	کیا آپ بچوں میں وقفے کے لیے ڈاکٹری طریقے اپنانے کا ارادہ رکھتی ہیں؟	[1]بالکل نہیں [2]کچھ خاص نہیں<br>[3]نہ ہاں نہ نہیں<br>[4]کچھ حد تک ہاں [5]مکمل طور پر ہاں	
	**مثبت رویہ**		
44	کیا آپ بچوں میں وقفے کے لئے ڈاکٹری طریقوں کے استعمال کو کار آمد سمجھتی ہیں؟	[1]بالکل نہیں [2]کچھ خاص نہیں<br>[3]نہ ہاں نہ نہیں<br>[4]کچھ حد تک ہاں [5]مکمل طور پر ہاں	

45	کیا آپ بچوں میں وقفے کے لئے ڈاکٹری طریقوں کے استعمال کو عقل مندانہ عمل سمجھتی ہیں؟	[1] بالکل نہیں<br>[2] کچھ خاص نہیں<br>[3] نہ ہاں نہ نہیں<br>[4] کچھ حد تک ہاں<br>[5] مکمل طور پر ہاں	
46	کیا آپ بچوں میں وقفے کے لئے ڈاکٹری طریقوں کے استعمال کو فائدہ مند سمجھتی ہیں؟	[1] بالکل نہیں<br>[2] کچھ خاص نہیں<br>[3] نہ ہاں نہ نہیں<br>[4] کچھ حد تک ہاں<br>[5] مکمل طور پر ہاں	
47	اگر قدرتی طور پر بچوں میں وقفہ نہ ہو تو ڈاکٹری طریقہ بچوں میں وقفے کے لیے موثر ہے؟	[1] بالکل نہیں<br>[2] کچھ خاص نہیں<br>[3] نہ ہاں نہ نہیں<br>[4] کچھ حد تک ہاں<br>[5] مکمل طور پر ہاں	
	**غلط فہمیاں اور خدشات**		
48	کیا آپ کے خیال میں بچوں میں وقفے کے لیے ڈاکٹری طریقہ استعمال کرنے سے آپ کی معمولی طبعیت خراب ہو سکتی ہے؟	[5] بالکل نہیں<br>[4] کچھ خاص نہیں<br>[3] نہ ہاں نہ نہیں<br>[2] کچھ حد تک ہاں<br>[1] مکمل طور پر ہاں	
49	کیا آپ کے خیال میں بچوں وقفے کے لیے ڈاکٹری طریقے استعمال کرنے سے آپ شدید بیمار ہو جائیں گے؟	[5] بالکل نہیں<br>[4] کچھ خاص نہیں<br>[3] نہ ہاں نہ نہیں<br>[2] کچھ حد تک ہاں<br>[1] مکمل طور پر ہاں	
50	آپ کے خیال میں کیا بچوں میں وقفے کے لیے ڈاکٹری طریقہ استعمال کرنا صحت کے لئے نقصان دے ہے؟	[5] بالکل نہیں<br>[4] کچھ خاص نہیں<br>[3] نہ ہاں نہ نہیں<br>[2] کچھ حد تک ہاں<br>[1] مکمل طور پر ہاں	
51	اگر آپ کو بچوں میں وقفے کا ڈاکٹری طریقہ استعمال کرنے سے کوئی بیماری ہو جائے تو کیا آپ کے خیال میں، آپ کو اس کے علاج پر بہت پیسے خرچ کرنے پڑیں گے؟	[5] بالکل نہیں<br>[4] کچھ خاص نہیں<br>[3] نہ ہاں نہ نہیں<br>[2] کچھ حد تک ہاں<br>[1] مکمل طور پر ہاں	
	**سمجھی اقدار**		
52	کیا آپ کے قریبی لوگ بچوں میں وقفے کے لئے ڈاکٹری طریقے کے استعمال کو اچھا سمجھتے ہیں؟	[1] بالکل نہیں<br>[2] کچھ خاص نہیں<br>[3] نہ ہاں نہ نہیں<br>[4] کچھ حد تک ہاں<br>[5] مکمل طور پر ہاں	
53	کیا آپ کے خیال میں آپ کے علاقے کے لوگ بچوں میں وقفے کے لیے ڈاکٹری طریقوں کی حمایت کرتے ہیں؟	[1] بالکل نہیں<br>[2] کچھ خاص نہیں<br>[3] نہ ہاں نہ نہیں<br>[4] کچھ حد تک ہاں<br>[5] مکمل طور پر ہاں	

54	کیا آپ کے خیال میں آپ کے قریبی لوگ ان عورتوں کو سیانا سمجھتے ہیں جو بچوں میں وقفے کے لیے ڈاکٹری طریقے استعمال کرتی ہیں؟	[1] بالکل نہیں [2] کچھ خاص نہیں<br>[3] نہ ہاں نہ نہیں<br>[4] کچھ حد تک ہاں [5] مکمل طور پر ہاں	
55	آپ کے قریبی / جان پہچان والے لوگوں کا خیال ہے آپ کو بچوں میں وقفے کے لیے ڈاکٹری طریقہ استعمال کرنا چاہیے؟	[1] بالکل نہیں [2] کچھ خاص نہیں<br>[3] نہ ہاں نہ نہیں<br>[4] کچھ حد تک ہاں [5] مکمل طور پر ہاں	
	**ذاتی قابو**		
56	کیا بچوں میں وقفے کے لئے ڈاکٹری طریقہ اپنانے کا فیصلہ آپ کے اختیار میں ہے؟	[1] بالکل نہیں [2] کچھ خاص نہیں<br>[3] نہ ہاں نہ نہیں<br>[4] کچھ حد تک ہاں [5] مکمل طور پر ہاں	
57	اگر آپ کو بچوں میں وقفے کے لیے ڈاکٹری طریقے استعمال کرنا ہو تو کیا اس میں آپ کو دشواری ہو گی؟	[1] بالکل نہیں [2] کچھ خاص نہیں<br>[3] نہ ہاں نہ نہیں<br>[4] کچھ حد تک ہاں [5] مکمل طور پر ہاں	
58	کیا آپ کے لئے، بچوں میں وقفے کے لئے ڈاکٹری طریقہ اپنانا آسان ہے؟	[1] بالکل نہیں [2] کچھ خاص نہیں<br>[3] نہ ہاں نہ نہیں<br>[4] کچھ حد تک ہاں [5] مکمل طور پر ہاں	

سیکشن چار: مشورات کی تشخیص، صوتی پیغام اور تحریری پیغام

اب میں آپ سے اُن صوتی پیغامات کے بارے میں پوچھوں گی جو آپ نے اپنے حمل کے دوران موصول کیے تھے

59	کیا آپ نے گزشتہ حمل کے دوران یا آخری زچگی کے بعد 42 دن کے اندر MSS سے کوئی صوتی پیغامات موصول کیے ہیں؟	[1] ہاں<br>[2] نہیں ←	← 70 پوچھیں
60	آپ نے کتنے پیغامات موصول کیے؟	پیغامات کی تعداد ☐☐<br>[98] یاد نہیں	
61	پیغامات کس زبان میں تھے؟	[1] اُردو [2] انگریزی [3] پنجابی<br>[4] سرائیکی [5] سندھی	
62	کیا پیغامات کو سمجھنا آسان تھا؟	[1] ہاں [2] نہیں	
63	پیغامات کا اوسط دورانیہ کتنا تھا؟	سیکنڈ ☐<br>منٹ ☐	
64	کیا MSS نے آپ کو اُن اوقات پر پیغامات بھیجے تھے جن میں آپ کے لیئے اُن کو سننا آسان تھا؟	[1] ہاں ←<br>[2] نہیں	← 66 پوچھیں

65	اگر نہیں تو پھر کس وقت آپ کو پیغامات بھیجنے چاہیئے تھے؟	وقت __:__	
66	کیاMSSکے مندرجہ ذیل اعمال سے آپ آرام دہ تھیں؟<br>ہاں نہیں<br>پیغامات کے اوقات ۱ ۲<br>پیغامات کا دورانیہ ۱ ۲<br>پیغامات کی زبان ۱ ۲<br>پیغامات کی تعداد ۱ ۲		
67	پیغامات کے ذریعہ MSS نے آپ کو کس قسم کی معلومات دی تھی؟<br><br>**[ایک سے زیادہ جوابات منتخب کرسکتے ہیں]**	۱-حمل کے دوران ڈاکٹری دوروں کی کم از کم تعداد<br>۲-حمل کے دوران ڈاکٹری دوروں کا وقت<br>۳-تشنج کے ٹیکوں کی تعداد حمل کے دوران<br>۴-حمل کے دوران غزائیت سپلمنٹ کے بارے میں معلومات<br>۵-برے حمل کے علامات<br>۶-برے حمل کی صورت میں کیا کرنا چاہیئے<br>۷-حمل کے دوران ایمرجنسی کی صورت میں کیا کرنا چاہیئے؟<br>۸-کیسے اور کب پیدائش کے لیئے تیاری کرنی چاہیے<br>۹-ضروری چیزوں کا انتظام جیسے پیسے، سفر، پیدائش کی جگہ، دیگر<br>۱۰-دودھ پلانے کے بارے میں معلومات<br>۱۱-بچوں کے حفاظتی ٹیکوں کے بارے میں معلومات<br>۱۲-نوزائیدہ بچے کو جو خطرات ہوتے ہیں<br>۱۳-بچوں کو کتنے حفاظتی ٹیکے لگنے چاہیئے<br>۱۴-نوازائیدہ بچے کی بنیادی ضروریات<br>۱۵-پیدائش کے بعد فیملی پلیننگ کی اہمیت<br>۱۶-فیملی پلیننگ حاصل کرنے کی جگہ<br>۱۷-پیدائش کے بعد ڈاکٹری دوروں کی کم از کم تعداد<br>۱۸-پیدائش کے بعد ڈاکٹری دوروں کا وقت<br>۱۹-پیدائش کے بعد کی پیچیدگیوں کی شناخت<br>۲۰-پیدائش کے بعد کے خطرات کا مشورہ<br>دیگر، وضاحت کریں<br>۱-__________<br>۲-__________<br>۳-__________	

		۴-__________	
68	کیا آپ MSS کی فراہم کردہ معلومات سے مطمئن ہیں؟	۱-مکمل طور پر غیر مطمئن<br>۲-کچھ حد تک غیر مطمئن<br>۳-نہ مطمئن نہ غیر مطمئن<br>۴-کچھ حد تک مطمئن<br>۵-مکمل طور پر مطمئن	
69	MSS کی فراہم کردہ معلومات کتنی کار آمد تھی؟	۱-انتہائی کار آمد<br>۲-کچھ حد تک کار آمد<br>۳-نہ کار آمد نہ غیر کار آمد<br>۴-کچھ حد تک غیر کار آمد<br>۵-انتہائی غیر کار آمد	
اب میں آپ سے اُن تحریری پیغامات کے بارے میں پوچھوں گی جو آپ نے اپنے حمل کے دوران موصول کیے تھے			
70	کیا آپ نے گزشتہ حمل کے دوران یا آخری زچگی کے بعد 42 دن کے اندر MSS سے کوئی تحریری پیغامات موصول کیے ہیں؟	[1] ہاں<br>[2] نہیں ←	←80 پوچھیں
71	آپ نے کتنے پیغامات موصول کیے؟	پیغامات کی تعداد [ ][ ]<br>[98] یاد نہیں	
72	پیغامات کس زبان میں تھے؟	[1] اُردو<br>[2] انگریزی<br>[3] پنجابی<br>[4] سرائیکی<br>[5] سندھی	
73	کیا پیغامات کو سمجھنا آسان تھا؟	[1] ہاں<br>[2] نہیں	
74	کیا MSS نے آپ کو اُن اوقات پر پیغامات بھیجے تھے جن میں آپ کے لیئے اُن کو پڑھنا آسان تھا؟	[1] ہاں ←<br>[2] نہیں	←76 پوچھیں
75	اگر نہیں تو پھر کس وقت آپ کو پیغامات بھیجنے چاہیئے تھے؟	وقت __:__	
76	کیا MSS کے مندرجہ ذیل اعمال سے آپ آرام دہ تھیں؟<br>ہاں / نہیں<br>پیغامات کے اوقات ۱ ۲<br>پیغامات کا دورانیہ ۱ ۲<br>پیغامات کی زبان ۱ ۲<br>پیغامات کی تعداد ۱ ۲		
77	پیغامات کے ذریعہ MSS نے آپ کو کس قسم کی معلومات دی تھی؟<br>**[ایک سے زیادہ جوابات منتخب کر سکتے ہیں]**	۱-حمل کے دوران ڈاکٹری دوروں کی کم از کم تعداد<br>۲-حمل کے دوران ڈاکٹری دوروں کا وقت<br>۳-تشنج کے ٹیکوں کی تعداد حمل کے دوران<br>۴-حمل کے دوران غزائیئت سپلمنٹ کے بارے میں معلومات<br>۵-برے حمل کے علامات<br>۶-برے حمل کی صورت میں کیا کرنا چاہیئے	

		۷-حمل کے دوران ایمرجنسی کی صورت میں کیا کرنا چاہیئے؟<br>۸-کیسے اور کب پیدائش کے لیئے تیاری کرنی چاہیے<br>۹-ضروری چیزوں کا انتظام جیسے پیسے، سفر، پیدائش کی جگہ، دیگر<br>۱۰-دودھ پلانے کے بارے میں معلومات<br>۱۱-بچوں کے حفاظتی ٹیکوں کے بارے میں معلومات<br>۱۲-نوزائیدہ بچے کو جو خطرات ہوتے ہیں<br>۱۳-بچوں کو کتنے حفاظتی ٹیکے لگنے چاہیئے<br>۱۴-نوازائیدہ بچے کی بنیادی ضروریات<br>۱۵-پیدائش کے بعد فیملی پلیننگ کی اہمیت<br>۱۶-فیملی پلیننگ حاصل کرنے کی جگہ<br>۱۷-پیدائش کے بعد ڈاکٹری دوروں کی کم از کم تعداد<br>۱۸-پیدائش کے بعد ڈاکٹری دوروں کا وقت<br>۱۹-پیدائش کے بعد کی پیچیدگیوں کی شناخت<br>۲۰-پیدائش کے بعد کے خطرات کا مشورہ<br>دیگر، وضاحت کریں<br>۱-__________<br>۲-__________<br>۳-__________<br>۴-__________	
78	کیا آپ MSS کی فراہم کردہ معلومات سے مطمئن ہیں؟	۱-مکمل طور پر غیر مطمئن<br>۲-کچھ حد تک غیر مطمئن<br>۳-نہ مطمئن نہ غیر مطمئن<br>۴-کچھ حد تک مطمئن<br>۵-مکمل طور پر مطمئن	
79	MSS کی فراہم کردہ معلومات کتنی کارآمد تھی؟	۱-انتہائی کارآمد<br>۲-کچھ حد تک کارآمد<br>۳-نہ کارآمد نہ غیر کارآمد<br>۴-کچھ حد تک غیر کارآمد<br>۵-انتہائی غیر کارآمد	
اب میں آپ سے اُن کالز کے بارے میں پوچھوں گی جو آپ نے اپنے حمل کے دوران موصول کیے تھے			
80	کیا آپ نے گزشتہ حمل کے دوران یا آخری زچگی کے بعد 42 دن کے اندر MSS سے کوئی کال موصول کیے ہیں؟	[1] ہاں<br>[2] نہیں ←	91 پوچھیں ←
81	آپ نے کتنی کالز موصول کی ہیں؟	پیغامات کی تعداد [ ][ ]<br>[98] یاد نہیں	

82	کال سینٹر کے نمائندہ نے کس زبان میں بات کی تھی؟	[1] اُردو [2] انگریزی [3] پنجابی<br>[4] سرائیکی [5] سندھی	
83	کیاMSS نے آپ کو اُن اوقات پر کال کی تھی جن میں آپ کے لیئے بات کرنا آسان تھا؟	[1] ہاں ←<br>[2] نہیں	← 85 پوچھیں
84	اگر نہیں تو پھر کس وقت آپ کو کال کرنی چاہیئے تھی؟	وقت __:__	
85	کیاMSS کے مندرجہ ذیل اعمال سے آپ آرام دہ تھیں؟		

	ہاں	نہیں
فون کال کے اوقات	۱	۲
فون کال کا دورانیہ	۱	۲
کال کی زبان	۱	۲
فون کال کی تعداد	۱	۲

86	کال کے ذریعہ MSS نے آپ کو کس قسم کی معلومات دی تھی؟<br><br>**[ایک سے زیادہ جوابات منتخب کرسکتے ہیں]**	۱-حمل کے دوران ڈاکٹری دوروں کی کم از کم تعداد<br>۲-حمل کے دوران ڈاکٹری دوروں کا وقت<br>۳-تشنج کے ٹیکوں کی تعداد حمل کے دوران<br>۴-حمل کے دوران غزائیئت سپلمنٹ کے بارے میں معلومات<br>۵-برے حمل کے علامات<br>۶-برے حمل کی صورت میں کیا کرنا چاہیئے<br>۷-حمل کے دوران ایمرجنسی کی صورت میں کیا کرنا چاہیئے؟<br>۸-کیسے اور کب پیدائیش کے لیئے تیاری کرنی چاہیے<br>۹-ضروری چیزوں کا انتظام جیسے پیسے، سفر، پیدائیش کی جگہ، دیگر<br>۱۰-دودھ پلانے کے بارے میں معلومات<br>۱۱-بچوں کے حفاظتی ٹیکوں کے بارے میں معلومات<br>۱۲-نوزائیدہ بچے کو جو خطرات ہوتے ہیں<br>۱۳-بچوں کو کتنے حفاظتی ٹیکے لگنے چاہیئے<br>۱۴-نوازائیداہ بچے کی بنیادی ضروریات<br>۱۵-پیدائیش کے بعد فیملی پلیننگ کی اہمیت<br>۱۶-فیملی پلیننگ حاصل کرنے کی جگہ<br>۱۷-پیدائیش کے بعد ڈاکٹری دوروں کی کم از کم تعداد<br>۱۸-پیدائیش کے بعد ڈاکٹری دوروں کا وقت<br>۱۹-پیدائش کے بعد کی پیچیدگیوں کی شناخت<br>۲۰-پیدائیش کے بعد کے خطرات کا مشورہ	

		دیگر، وضاحت کریں<br>۱-____________________<br>۲-____________________<br>۳-____________________<br>۴-____________________	
87	کیا آپ MSS کی فراہم کردہ معلومات سے مطمئن ہیں؟	۱-مکمل طور پر غیر مطمئن ۲-کچھ حد تک غیر مطمئن<br>۳-نہ مطمئن نہ غیر مطمئن ۴-کچھ حد تک مطمئن<br>۵-مکمل طور پر مطمئن	
88	کیا آپ MSS کے کال سینٹر کے نمائندہ کے رویے سے مطمئن تھیں؟	۱-انتہائی کارآمد ۲-کچھ حد تک کارآمد<br>۳-نہ کارآمد نہ غیر کارآمد ۴-کچھ حد تک غیر کارآمد<br>۵-انتہائی غیر کارآمد	
89	کیا آپ MSS کے کال سینٹر کا نمائندہ آپ کے مژلات کو سمجھ پایا تھا؟	[1]ہاں [2]نہیں	
90	MSS کی فراہم کردہ معلومات کتنی کارآمد تھی؟	۱-انتہائی کارآمد ۲-کچھ حد تک کارآمد<br>۳-نہ کارآمد نہ غیر کارآمد ۴-کچھ حد تک غیر کارآمد<br>۵-انتہائی غیر کارآمد	
اب میں آپ سے کالز، تحریری پیغامات اور صوتی پیغامات کے پورے تجربے کے بارے میں پوچھوں گی			
91	کیا چیزیں سیکھنا آسان تھیں؟	[1]ہاں [2]نہیں	
92	**[پوچھیں کہ جواب دہندہ نے تحریری اور صوتی پیغامات موصول کیے تھے، اگر نہیں تو سوال نمبر 435 پوچھیں]**<br>کچھ عورتوں نے تحریری اور صوتی پیغامات موصول کیے تھے۔ ان پیغامات کے ذریعہ عورتیں مخصوص معلومات حاصل کر سکتی ہیں اُن پیغامات کا جواب دے کر۔ تو کیا آپ بھی جواب دینے کی سہولت کا استعمال کر کے نمائندہ سے بات کرنا چاہیں گیں؟	[1]ہاں [2]نہیں	
93	اگر آپ کسی اور ذریعے سے معلومات حاصل کر سکتیں تو آپ کونسا ذریعہ پسند کرتیں؟<br>**[ایک سے زیادہ جوابات منتخب کر سکتے ہیں]**	۱-تحریری پیغام ۲-صوتی پیغام<br>۳-فون کال ۴-کوئی بھی نہیں	
94	کیا آپ کے پاس کوئی مشورے ہیں ہمارے پروگرام کیلیے؟	[1]ہاں<br>[2]نہیں ←	←96 پوچھیں

95	اگر ہاں، تو آپ کیا مشورہ دینا چاہیں گیں؟	۱-__________<br>۲-__________<br>۳-__________<br>۴-__________	
96	کیا اس تحقیق میں شمولیت کرنے سے آپ کو کوئی بُرا تجربہ ہوا؟	[1]ہاں<br>[2]نہیں ←	←100 پوچھیں
97	اگر ہاں، تو کیا ہوا؟	[1]ڈرایا اور ہتھیار کا استعمال کیا<br>[2]تھپڑ مارا، دھکا، کوئی زخم نہیں آیا اور تھوڑی دیر تک رہا<br>[3]مُکا، گھوسا مارا، خراش آئی، کٹ لگا اور مسلسل درد رہا<br>[4]مارا، گھیرا زخم آیا، جل گیا، ہڈی ٹوٹ گئی<br>[5]سر پر چوٹ آئی، اندرونی چوٹ آئی، زخم پڑ گیا<br>[6]ہتھیار کا استعمال کیا	
98	اگر ہاں، تو کس نے کیا؟	[1]شوہر<br>[2]سرال والوں نے<br>[3]دیگر	
99	کتنی بار ایسا ہوا؟	تعداد__________	
100	کیا آپ MSS سے مزید مشورات یا معلومات موصول کرنا پسند کریں گیں؟	[1]ہاں<br>[2]نہیں ←	←102 پوچھیں
101	آپ مزید کیا جاننا چاہیں گیں؟	۱-__________<br>۲-__________<br>۳-__________<br>۴-__________	
102	کیا آپ اپنی دوستوں کو مشورہ دیں گیں اِس پروگرام میں حصّہ لینے کیلیے؟	[1]ہاں ←<br>[2]نہیں	←104 پوچھیں
103	اگر نہیں تو کیوں نہیں؟	۱-__________<br>۲-__________<br>۳-__________<br>۴-__________	

سیکشن پانچ: سماجی تعاون

شرکت کنندہ کو پڑھ کر سنائیں: ہم ان لوگوں کے بارے میں جاننا چاہتے ہیں جو آپ کے لئے معاون و مدد گار ہیں۔ معاون و مدد گار سے مراد وہ شخص ہے جو آپ کی مدد کرے، آپ کی بات سنے یا مشکل میں آپ کا ساتھ دے / آپ کے ساتھ کھڑا ہو۔ ہم ایک ایک کر کے مختلف لوگوں کے حوالے سے سوالات پوچھیں گے۔ آپ کو فیصلہ کرنا ہے کہ وہ شخص یا گروہ آپ کے لئے کس حد تک معاون و مدد گار ہے۔

برائے مہربانی متعلقہ جواب پر دائیرہ لگائیں۔

104	آپ کے شوہر آج کل آپ کے لیے کس حد تک آپکا ساتھ دیتے ہیں یا آپکی مدد کرتے ہیں؟	[0] بلکل نہیں [1] کچھ حد تک [2] بہت زیادہ	
105	کیا آپ کا 18 سال سے بڑا کوئی بچہ ہے؟	[1] ہاں<br>[2] نہیں	← 107 پوچھیں
106	آپ کا بڑا بچہ (یا بچے) آج کل آپ کا کس حد تک ساتھ دیتے ہیں یا مدد کرتے ہیں؟	[0] بلکل نہیں [1] کچھ حد تک [2] بہت زیادہ	
107	کیا آپ کے والدین (والد / والدہ) حیات [زندہ] ہیں؟	[1] ہاں<br>[2] نہیں	← 109 پوچھیں
108	آپ کے والدین (والد / والدہ) آج کل آپ کا کس حد تک ساتھ دیتے ہیں یا مدد کرتے ہیں؟	[0] بلکل نہیں [1] کچھ حد تک [2] بہت زیادہ	
109	کیا آپ کے بہن بھائی ہیں؟	[1] ہاں<br>[2] نہیں	← 111 پوچھیں
110	آپ کے بہن بھائی آج کل آپ کا کس حد تک ساتھ دیتے یا مدد کرتے ہیں؟	[0] بلکل نہیں [1] کچھ حد تک [2] بہت زیادہ	
111	کیا آپ کے چچا، پھپو، ماموں، خالا اور ان کے بچے ہیں؟	[1] ہاں<br>[2] نہیں	← 113 پوچھیں
112	آپ کے چچا، پھپو، ماموں، خالا اور ان کے بچے آج کل آپکا کس حد تک ساتھ دیتے ہیں یا مدد کرتے ہیں؟	[0] بلکل نہیں [1] کچھ حد تک [2] بہت زیادہ	
113	کیا آپ کے سسرالی رشتہ دار ہیں مثلاً ساس، سسر، نند، دیور، جیٹھ؟	[1] ہاں<br>[2] نہیں	← 115 پوچھیں
114	آپ کے سسرالی رشتہ دار آج کل آپکا کس حد تک ساتھ دیتے ہیں یا مدد کرتے ہیں؟	[0] بلکل نہیں [1] کچھ حد تک [2] بہت زیادہ	
115	کیا آپ کے (رشتہ داروں کے علاوہ) ہمسائے / پڑوسی ہیں؟	[1] ہاں<br>[2] نہیں	← 117 پوچھیں
116	آپ کے ہمسائے / پڑوسی آج کل کس حد تک آپ کا ساتھ	[0] بلکل نہیں [1] کچھ حد تک [2] بہت زیادہ	

	دیتے ہیں یا مدد کرتے ہیں؟		
117	کیا کوئی سرکاری یا غیر سرکاری ادارے کی ورکر آپ کے گھر آتی جاتی ہے؟	[1] ہاں<br>[2] نہیں ←	122 پوچھیں
118	سرکاری یا غیر سرکاری ادارے کی ورکر آج کل آپ کے لئے کس حد تک مدد گار ہے؟<br>ہر ایک کے نام اور عہدے کے ساتھ بتائیں کہ وہ کس حد تک اور مدد گار ہے:		
119	نام اور عہدہ 1:____________	[0] بلکل نہیں [1] کچھ حد تک [2] بہت زیادہ	
120	نام اور عہدہ 2:____________	[0] بلکل نہیں [1] کچھ حد تک [2] بہت زیادہ	
121	نام اور عہدہ 3:____________	[0] بلکل نہیں [1] کچھ حد تک [2] بہت زیادہ	
122	کیا آپ کسی مقامی فلاحی کمیٹی / مذہبی مجالس کا حصہ ہیں؟	[1] ہاں<br>[2] نہیں ←	124 پوچھیں
123	مقامی فلاحی کمیٹی / مذہبی مجالس کے افراد آج کل آپ کے لیے کس حد تک معاون اور مدد گار ہیں؟	[0] بلکل نہیں [1] کچھ حد تک [2] بہت زیادہ	
124	کیا آپ کی دوست یا سہیلیاں ہیں؟	[1] ہاں<br>[2] نہیں ←	126 پوچھیں
125	آپ کی دوست یا سہیلیاں آج کل آپ کی کس حد تک مدد کرتی ہیں یا ساتھ دیتی ہیں؟	[0] بلکل نہیں [1] کچھ حد تک [2] بہت زیادہ	
126	کیا آپ کے پاس کوئی ایک ایسا خاص شخص ہے جس پر آپ بھروسہ کر سکیں اور اسے اپنے ذاتی مسائل بتا سکیں؟	[1] ہاں<br>[2] نہیں ←	128 پوچھیں
127	اگر ہاں تو اوپر بتائے گئے لوگوں میں سے وہ شخص کون ہے؟	شخص کا رشتہ درج کریں (جیسے شوہر / بھائی / بہن / ساس وغیرہ)<br>رشتہ:____________	

سیکشن چھ: گھریلو تشدد

انٹرویو کرنے والے کے لیے ہدایات: معائنہ کریں کہ جس جگہ آپ بیٹھی ہیں یا آپ کے پاس کی جگہیں جہاں آپ کی آواز پہنچ سکتی ہو، وہاں کوئی اور موجود نہ ہو۔ رازداری کو برقرار رکھنے کے لیے اس بات کی یقین دہانی کریں کہ کوئی بھی فرد اتنے قریب نہ ہو کہ وہ آپکے سوال وجواب کو سن سکے۔ اگر آس پاس اس عمر کے بچے ہیں جو آپکی باتوں کو سمجھنے کی صلاحیت رکھتے ہیں، انھیں شائستگی سے جانے کا کہیں۔ صرف چھوٹے بچے جو آپ کی باتوں کو سمجھنے کے قابل نا ہوں، وہ بات چیت کے دوران آپ کے ہمراہ رہ سکتے ہیں۔ اُس وقت تک سوالات نہ پوچھیں، جب تک اس بات کی یقین دہانی نہ کر لیں کے آپ کے آس پاس کوئی نہیں ہے۔ اس معلومات کی اہمیت کو نظر انداز نہیں کیا جا سکتا، آپ کو رازداری رکھنے کے لیے ہر وہ عمل کرنا ہے جس کے بعد آپ سوال وجواب شروع کر سکیں۔ اگر آپ رازداری کو برقرار رکھنے میں ناکام رہیں گی تو بہت سی اہم معلومات حاصل نہیں کر پائینگی۔

جوابدہندہ کے لیئے پڑھیے: اب میں آپ سے خواتین کی زندگی کے کچھ اہم پہلوؤں کے بارے میں سوالات کرنا چاہتی ہوں۔ ہو سکتا ہے ان میں سے کچھ سوالات آپ کو بہت ہی

ذاتی نوعیت کے لگیں۔ تاہم آپ کے جوابات پاکستان میں موجود خواتین کے حالات کو سمجھنے میں بے انتہا مددگار ثابت ہوں گے۔ میں آپ کو اس بات کی یقین دہانی کرواتی ہوں کہ آپ کے جوابات مکمل طور پر خفیہ رکھے جائیں گے اور ان کا ذکر کسی سے نہیں کیا جائے گا اور آپ کے گھرانے میں کسی کو یہ نہیں معلوم ہو گا کہ آپ سے کس قسم کے سوالات پوچھے گئے ہیں۔

128	کیا آپ کو کبھی بھی اپنے شوہر یا قریبی عزیز کی طرف سے جسمانی یا جذباتی تشدد کا نشانہ بنایا گیا؟	[1]ہاں<br>[2]نہیں	132 پوچھیں ◄
129	گزشتہ 6 ماہ کے دوران، آپکو کسی نے مارا، تھپڑ مارا، ٹھوکر ماری یا کوئی جسمانی تشدد کیا؟	[1]ہاں<br>[2]نہیں	132 پوچھیں ◄
130	اگر ہاں، تو کس نے؟	[1]شوہر [2]سسرال [3]کوئی اور	
131	کتنی دفعہ تشدد کیا؟	تعداد ☐☐	
132	جب سے آپ حاملہ ہوئی، آپکو کسی نے مارا، تھپڑ مارا، ٹھوکر ماری یا کوئی جسمانی تشدد کیا؟	[1]ہاں<br>[2]نہیں	138 پوچھیں ◄
133	اگر ہاں، تو کس نے؟	[1]شوہر [2]سسرال [3]کوئی اور	
134	کتنی دفعہ تشدد کیا؟	تعداد ☐☐	
135	آپ کے جسم کے کس حصے پر سب سے زیادہ تشدد کیا گیا؟<br>**[صرف ایک جواب ممکن ہے، ایک سے زیادہ جواب آنے کی صورت میں سب سے اہم جواب نوٹ کریں]**	[1]سر پر<br>[2]چہرے پر<br>[3]چھاتی / سینہ پر<br>[4]پیٹ پر<br>[5]پیٹھ پر / کمر کے اوپری حصہ پر<br>[6]پشت پر، کمر کے نچلے / جسم کے پچھلے حصہ پر<br>[7]ٹانگ / گھٹنے پر	
136	تشدد کس طرح کیا گیا یعنی تشدد کے لئے کس قسم کا اوزار / ہتھیار کا استعمال کیا گیا؟	[1]ہاتھ / پیر کا استعمال کیا<br>[2]ہتھیار کا استعمال جیسے چھری / لکڑی / برتن / ڈنڈا / پایپ وغیرہ<br>دیگر (وضاحت کریں)__________	
137	**تشدد کی نوعیت کیا تھی یعنی، کیا تشدد کرنے سے:**	**ہاں** / **نہیں**	
	[1]چوٹ یا تھوڑے وقت تک درد رہا۔	1 / 2	
	[2]زخم، کٹ پڑ گیا یا مسلسل درد رہا۔	1 / 2	
	[3]گہرا زخم جس سے خون آیا یا ہڈی ٹوٹ گئی۔	1 / 2	
	[4]اندرونی چوٹ، مستقل چوٹ رہی۔	1 / 2	
138	گزشتہ 6 ماہ کے دوران، کبھی آپ کے شوہر نے آپ کو زبردستی جسمانی تعلق قائم کرنے کے لیے زور دیا؟	[1]ہاں<br>[2]نہیں	140 پوچھیں ◄
139	کتنی دفعہ تشدد کیا؟	تعداد ☐☐	

140	کیا آپ اپنے شوہر سے خوفزدہ رہتے ہو؟	[1]ہاں [2]نہیں	
	**آپ کے وقت کا شکریہ**		
